# Iterative Most-Likely Point Registration (IMLP): A Robust Algorithm for Computing Optimal Shape Alignment

**DOI:** 10.1371/journal.pone.0117688

**Published:** 2015-03-06

**Authors:** Seth D. Billings, Emad M. Boctor, Russell H. Taylor

**Affiliations:** 1 Department of Computer Science, Johns Hopkins University, Baltimore, MD, United States of America; 2 Division of Medical Imaging Physics, Department of Radiology, Johns Hopkins Medical Institutions, Baltimore, MD, United States of America; 3 Department of Electrical & Computer Engineering, Johns Hopkins University, Baltimore, MD, United States of America; Bangladesh University of Engineering and Technology, BANGLADESH

## Abstract

We present a probabilistic registration algorithm that robustly solves the problem of rigid-body alignment between two shapes with high accuracy, by aptly modeling measurement noise in each shape, whether isotropic or anisotropic. For point-cloud shapes, the probabilistic framework additionally enables modeling locally-linear surface regions in the vicinity of each point to further improve registration accuracy. The proposed Iterative Most-Likely Point (IMLP) algorithm is formed as a variant of the popular Iterative Closest Point (ICP) algorithm, which iterates between point-correspondence and point-registration steps. IMLP’s probabilistic framework is used to incorporate a generalized noise model into both the correspondence and the registration phases of the algorithm, hence its name as a most-likely point method rather than a closest-point method. To efficiently compute the most-likely correspondences, we devise a novel search strategy based on a principal direction (PD)-tree search. We also propose a new approach to solve the generalized total-least-squares (GTLS) sub-problem of the registration phase, wherein the point correspondences are registered under a generalized noise model. Our GTLS approach has improved accuracy, efficiency, and stability compared to prior methods presented for this problem and offers a straightforward implementation using standard least squares. We evaluate the performance of IMLP relative to a large number of prior algorithms including ICP, a robust variant on ICP, Generalized ICP (GICP), and Coherent Point Drift (CPD), as well as drawing close comparison with the prior anisotropic registration methods of GTLS-ICP and A-ICP. The performance of IMLP is shown to be superior with respect to these algorithms over a wide range of noise conditions, outliers, and misalignments using both mesh and point-cloud representations of various shapes.

## Introduction

The need to co-align multiple representations of a shape or environment is a problem commonly encountered in numerous fields such as robotics, computer vision, and computer-integrated medical procedures. An early method devised to address this problem is the widely popular Iterative Closest Point (ICP) algorithm [1]. ICP operates by decomposing one of the shapes to be registered (the source shape) into a set of points (if not already in point form) and then computing a spatial transformation to align these points to the second shape (the target shape). The registration is performed through a two-step iterative procedure that first computes matching points on the target shape that lie closest to each point of the source shape (the correspondence phase) and then computes the rigid-body spatial transformation, composed of a rotation and translation, that minimizes the sum of square distances between the matched points (the registration phase). This process iterates until the two shapes converge upon a stable alignment. An important implementation concern regards efficient techniques to compute matches in the correspondence phase; the standard approach for closest-point matching is to use a KD tree [2].

Following the introduction of ICP by Besl and McKay [1], many variants of the standard procedure have been proposed. Chen and Medioni [3] minimize point-to-plane square distances between the source points and planes tangent to the target surface at the corresponding target points. They demonstrate the usefulness of this method for registration of range images. Zhang [4] presents a robust ICP variant that incorporates robust statistics and adaptive thresholding to handle outliers and occlusions in the correspondence phase. Maurer et al. [5] introduce weighting terms in the registration phase for each point-pair, which they use for outlier rejection and for normalization of non-uniform point densities when registering head segmentations from medical images. Others have sought to improve correspondence selection by augmenting the match metric with additional information besides distance. Sharp et al. [6], for example, use feature invariants such as curvature to refine match selection. In [7] Armesto et al. present an alternate metric-based distance function for the scan-matching problem in mobile robotics, which takes into account both translation and rotation error of the sensor. Their work is based on extending the 2D metric-based ICP (MbICP) method of Minguez et al. [8] to the 3D case. An interesting approach by Fitzgibbon [9] directly minimizes a model-data error function using the nonlinear Levenberg-Marquardt algorithm while providing robust estimation via a Huber kernel. This approach is made efficient by pre-computing a distance transform on the target shape.

More recently, researchers have investigated probabilistic methods to improve upon the accuracy and flexibility of the standard ICP algorithm through incorporation of generalized noise models. In contrast, the standard ICP method and most variants implicitly assume an isotropic noise model. Estépar et al. [10] introduce the robust Generalized Total-Least-Squares ICP (GTLS-ICP) algorithm for registration problems in medical imaging, which incorporates a generalized total-least-squares framework within the registration phase of the algorithm to account for anisotropic noise in the measured data points. Segal et al. [11] later employ a similar framework for their Generalized ICP (GICP) algorithm; instead of using the probabilistic framework to model measurement noise, however, they structure the noise model to approximately minimize a plane-to-plane square distance metric, which they demonstrate by range image registration to have an accuracy advantage compared to the point-to-plane method of Chen and Medioni [3]. The methods of Estépar et al. and Segal et al. both follow standard ICP procedure in the correspondence phase by using closest points as the match criteria. Maier-Hein et al. [12] later introduce Anisotropic ICP (A-ICP), which primarily extends the works of Estépar et al. and Segal et al. by modifying the match criteria of the correspondence phase to minimize a Mahalanobis-distance metric defined by the covariances of the noise model. In lieu of an efficient method to compute such matches, A-ICP follows a procedure of first computing an initial registration using standard ICP and then continuing the registration with A-ICP while enforcing a user-defined bound on the search distance in the correspondence phase to reduce runtime. Moghari and Abolmaesumi [13] propose an ICP-like method based on the Unscented Kalman Filter algorithm, which is also able to account for anisotropic measurement error. Their method, which was further evaluated in [14], is an improvement over the Extended Kalman Filter algorithm of Pennec and Thirion [15].

Other authors incorporate probabilistic methods in a different manner by using soft matching, where each point in the source shape is matched to every point in the target shape (rather than just one point) with a varying weight or probability associated to each pairing. Early works pioneering this approach were presented by Gold et al. [16] using the softassign technique for matching and by Chui and Rangarajan [17] (TPS-RMP) and Granger and Pennec [18] (EM-ICP) using Gaussian mixture models (GMMs) optimized within an expectation maximization (EM) framework. In addition to rigid registration, Chui and Rangarajan present a non-rigid method based on thin-plate splines. An alternate consistent and symmetric approach for non-rigid registration based on EM-ICP is given by Combès and Prima [19]. A modern variant of the EM-based methods called Coherent Point Drift (CPD) was presented by Myronenko and Song [20], in which they present a closed-form M-step solution for the rigid-body alignment problem and use Gaussian radial basis functions for the non-rigid alignment problem. The CPD algorithm treats one point cloud as the centroids of a GMM, which is aligned by maximum likelihood to a data set represented by the second point cloud. Robustness to outliers is enabled by additionally matching each point to the background using an outlier weighting parameter. An alternate approach presented by Tsin and Kanade [21] treats each set of points as separate kernel densities formed from Gaussian kernel functions centered at each point; the registration is computed by maximizing a kernel correlation (KC) metric between the two densities. Jian and Vemuri [22] present a similar idea for rigid and non-rigid registration by forming GMMs from each point set and minimizing the L2 distance between the Gaussian mixtures. While soft-match variants of ICP tend to achieve higher accuracy and to have wider basins of convergence towards the global optimum, these algorithms also tend to be less efficient than the single-match variants due to the exhaustive point pairings.

In this paper, we propose a new variant of ICP, called the Iterative Most-Likely Point (IMLP) algorithm, which incorporates a probabilistic framework similar to the algorithms of [10–12]. Overall, our method is most similar to A-ICP [12] and likewise incorporates a generalized noise model within both the registration and correspondence phases of the algorithm. A notable difference of our method is that point correspondences are computed to maximize the match likelihood function under the assumed multivariate Gaussian noise model (thus its name), whereas A-ICP computes correspondences to minimize a square Mahalanobis-distance metric. As we will show, these approaches are not equivalent and this difference in match criteria can dramatically impact the accuracy of the computed registration. IMLP also includes distinct approaches for registering shapes of partial overlap and for handling outliers, which is based in part on a dynamic updating of the noise model to account for uncertainty in the matches. As an important implementation concern, we present a novel scheme to efficiently compute the most-likely matches, which enables IMLP to run efficiently. In contrast, the implementation presented for A-ICP relies on pre-registration by an alternate algorithm. Finally, we present a new solution to the generalized total-least-squares (GTLS) optimization problem of the registration phase that is based on a Gauss-Newton approach and that has both speed and accuracy advantages compared to prior published solutions for this problem, while being straightforward to implement. The following paragraphs involve summary discussions of these contributions in further detail.

In this paper, we devise a new search strategy for computing point correspondences under an anisotropic distance criterion, which is based on a modified principal direction (PD)-tree search. A description of the standard PD-tree search technique is found in [23]. While our approach was devised for the most-likely match criterion of IMLP, it is equally applicable to the Mahalanobis-distance match criterion of A-ICP. Our method is efficient and guarantees that the best match, as defined by the match criterion, is always selected from the target shape. The PD tree, also known as the PCA or covariance tree, is similar in concept to the KD tree except that the local coordinate systems assigned to each node of the tree do not have constrained orientations with respect to a global coordinate frame. Williams et al. [24] previously investigated using a PD tree for the problem of closest-point matching.

The efficient correspondence search introduced in this paper is an essential element of the IMLP algorithm, as the primary computational bottleneck for ICP-based methods occurs at the correspondence search. Having an efficient search strategy is therefore critical for the performance and usefulness of these algorithms in practice. As was already mentioned, among the closely related prior works GTLS-ICP and GICP address the issue by simply using closest-point matching, which has an efficient implementation based on the KD-tree data structure [2]. The prior work of A-ICP [12], which does not directly look up matches from a KD tree due to its anisotropic match criteria, addresses the problem of efficiency by first registering the shapes with an alternative ICP-based algorithm and then performing a follow-up registration using A-ICP. In addition, A-ICP imposes a distance bound on the search radius to limit the pool of match candidates for each sample point, with the pool of match candidates being formed using a KD tree. One drawback of this approach is that locating the best match, as defined by the match criteria, cannot be guaranteed. Further, the search within the pool of match candidates is performed exhaustively.

Besides the need for efficient matching in the correspondence phase, a second implementation concern for IMLP regards solving the optimization problem of the registration phase, which computes the rigid-body transformation that optimally aligns the corresponding point sets obtained from the correspondence phase, while taking into account the generalized noise model. Various closed-form solutions for minimizing the isotropic square-distance metric of the standard ICP algorithm have been presented by Horn [25], Arun et al. [26], and Walker et al. [27], which have standard least-squares solutions. The probability framework incorporated by IMLP and by related anisotropic methods leads to a nonlinear generalized total-least-squares (GTLS) optimization over the transformation parameters in the registration phase, for which no closed-form solution is known. Solving the GTLS problem thus requires more complex iterative methods of nonlinear optimization.

As alluded to above, the prior algorithms of Estépar et al. (GTLS-ICP) [10], Segal et al. (GICP) [11], and Maier-Hein et al. (A-ICP) [12] share in common with IMLP the same GTLS problem for computing optimal alignment between corresponding point sets. Estépar et al. present an ad-hoc solution that incorporates, as a component, the iterative GTLS rotation estimation method of Ohta and Kanatani [28], which is based on Kanataniâs renormalization technique [29]. Kanataniâs method solves the problem of computing rotation when translation is known using a quaternion parameterization of the rotation matrix. Estépar et al. extend this solution to solve the parallel problem of computing translation when rotation is known. Their approach for solving the full GTLS rigid-body alignment problem is then a dual-iterative one that first computes rotation assuming known translation and then computes translation assuming known rotation. This process iterates until both estimates converge. Another solution, which to our knowledge has not been applied in an ICP-based context, is presented in a paper by Matei and Meer [30] regarding their heteroscedastic errors-in-variables (HEIV) estimator. The HEIV estimator is a general-purpose method for solving a wide range of problems in computer vision through iterative solutions of a generalized eigenvector problem. The GTLS rigid-body point-set alignment problem is presented as an example application of this technique in [30]. Their solution is similar to the renormalization approach followed in [28] in that both approaches involve solving eigenvalue problems and both use a quaternion parameterization for rotation. Rather than follow an ad-hoc approach, Segal et al. apply a generic conjugate-gradient solver to optimize the GTLS cost function of GICP, in which rotation is parameterized as three Euler angles (as referenced in distributed source code). Maier-Hein et al. employ an ad-hoc approach presented by Balachandran and Fitzpatrick in [31] and further analyzed in [32], which simultaneously solves for rotation and translation by successive linearization of the rotation matrix using a skew-matrix approximation for small rotation. One limitation of this method is that anisotropic noise is assumed for only one of the point sets, which may lead to inaccurate results when both point sets have anisotropic error distributions.

In this paper, we introduce an alternative approach for solving the GTLS problem of aligning corresponding point sets that supports anisotropic noise in both sets of points. Our approach is based on a modified Gauss-Newton framework that is efficient, stable, and simple to implement using a standard least-squares solver. As demonstrated in the Results and Discussion section of this paper, the proposed Gauss-Newton-based approach has advantages compared to the prior ad-hoc methods of Estépar et al. and Balachandran and Fitzpatrick in terms of accuracy, speed, and stability. A benefit of our method, and of the prior ad-hoc methods, is that only a standard least-squares solver is required for its implementation; thus, the software dependency of a nonlinear optimization library is avoided.

The remainder of this paper is structured as follows. A Background subsection completes the Introduction section by providing an algorithmic summary of the standard ICP algorithm as added background for the reader. The Methods section presents the new algorithms proposed in this paper. It begins by introducing the proposed IMLP algorithm at a high level and follows with subsections that detail our approach for each sub-phase of the algorithm, i.e. our approach to efficiently compute the most-likely matches in the correspondence phase and our approach to solve the GTLS problem of registering corresponding point sets in the registration phase. The Results and Discussion section presents an evaluation of our proposed algorithms with respect to a large body of prior works. Finally, in the Conclusions section we summarize our contributions and present our concluding remarks.

### Background

#### Iterative Closest Point (ICP) Algorithm

Let $X=\{{\vec{x}}_{i}\}$ be a set of points representing the source shape. Suppose the target shape is represented by Ψ, and let $Y=\{{\vec{y}}_{i}\}$ be a set of points chosen from this target shape that are in correspondence with (i.e. matched to) the points in *X*. As previously described, ICP proceeds by iterating between two key steps:

1. Compute a set of correspondences *Y* from the target shape using the closest-point operator C_CP_ (1).
  $$\begin{array}{c} {\text{C}}_{\text{CP}}\left(\vec{x},\Psi \right)=\underset{\vec{y}\;\in \;\Psi }{\text{argmin}}{∥\vec{y}-\vec{x}∥}_{2} \end{array}$$ (1)
2. Compute the rigid-body spatial transformation, comprised of rotation *R* and translation $\vec{t}$ and applied to the source shape *X*, that minimizes the sum of square distances between corresponding points (2).
  $$\begin{array}{c} {\text{E}}_{\text{LS}}\left(X,Y\right)=\min_{\left[R,\vec{t}\;\right]}\sum_{i=1}^{n}{∥{\vec{y}}_{i}-R{\vec{x}}_{i}-\vec{t}\;∥}_{2}^{2} \end{array}$$ (2)

The first step may be computed efficiently using a KD-tree search. The second step has a closed-form solution computable via Arun’s method [26]. Algorithm 1 provides a summary of the ICP algorithm.

**Algorithm 1.** Iterative Closest Point (ICP)

**input**: Source shape as point cloud: $X=\{{\vec{x}}_{i}\}$

    Target shape: Ψ

    Initial transformation estimate: $\left[R_{0},{\vec{t}}_{0}\right]$

**output**: Final transformation $\left[R,\vec{t}\right]$ that aligns the shapes *X* and Ψ

**1** Initialize transformation: $\left[R,\vec{t}\right]\leftarrow \left[R_{0},{\vec{t}}_{0}\right]$

**2** **while** *not converged* **do**

**3**  Compute closest-point correspondences $y=\{{\vec{Y}}_{i}\}$:

${\vec{y}}_{i}\leftarrow {\text{C}}_{\text{cp}}(R{\vec{x}}_{i}+\vec{t},\Psi )$

**4**  Update the transformation to minimize E_LS_(*X, Y*):

$[R,\vec{t}\;]\leftarrow \underset{\left[R,\vec{t}\;\right]}{\text{argmin}}\sum_{i=1}^{n}\|{\vec{y}}_{i}-R{\vec{x}}_{i}-\vec{t}\;{\|}_{2}^{2}$

**5 end**

## Methods

In this section, we present the proposed Iterative Most-Likely Point (IMLP) algorithm. We first provide an overview of the method, followed by subsections detailing our approach to each sub-phase of the algorithm, i.e. detailing our efficient search strategy for computing most-likely matches in the correspondence phase and detailing our method for solving the alignment of corresponding point sets in the registration phase.

Source code for the IMLP algorithm and the experimental data used in this paper are provided for download at: https://github.com/sbillin/IMLP.

### Iterative Most-Likely Point (IMLP) Algorithm

The probabilistic framework of IMLP incorporates a generalized noise model that accounts for anisotropic errors in both the source- and target-point positions. The errors on the measurements of these points are assumed to be independent, zero-mean, multivariate, Gaussian distributed. Thus, treating correspondence as a parameter to be estimated, the likelihood that a transformed source point $\left(R\vec{x}+\vec{t}\right)$ corresponds to a specific target point $\vec{y}$ is defined as

$$\begin{array}{ccc} {\text{L}}_{\text{match}}(\vec{x},\vec{y},M_{\text{x}},M_{\text{y}},R,\vec{t}\;) & = & \\ & & \frac{1}{\sqrt{{\left(2\pi \right)}^{3}|RM_{\text{x}}R^{T}+M_{\text{y}}|}}e^{-\frac{1}{2}{\left(\vec{y}-R\vec{x}-\vec{t}\;\right)}^{T}{\left(RM_{\text{x}}R^{T}+M_{\text{y}}\right)}^{-1}\left(\vec{y}-R\vec{x}-\vec{t}\;\right)} \end{array}$$ (3)

where *M*_x_ and *M*_y_ are known covariance matrices describing the noise-model distributions of the source point $\vec{x}$ and target point $\vec{y}$ , respectively. We refer to (3) as the “match-likelihood function”.

The match-likelihood function establishes the probabilistic foundation for IMLP. In the correspondence phase of the algorithm, a match for each source point is selected from the target shape to maximize the match-likelihood function while considering the transformation parameters *R* and $\vec{t}$ as known. In the registration phase of the algorithm, the transformation parameters *R* and $\vec{t}$ are then updated to maximize the total likelihood over all matched points while considering the matches as known. It is interesting to note that for the case of uniform, isotropic covariances then maximizing the match-likelihood function reduces to minimizing the square match distances, which is the criteria used by standard ICP. Algorithm 2 provides a summary of the IMLP algorithm, to which we refer back repeatedly in the discussion that follows.

**Algorithm 2.** Iterative Most-Likely Point (IMLP)

**input**: Source shape as point cloud: $X=\{{\vec{x}}_{i}\}$

    Target shape: Ψ

    Measurement-error covariances: *M*_X_ = {*M*_X*i*_}, *M*_Ψ_

    Surface-model covariances: *M*_SX_ = {*M*_Sx*i*_}, *M*_SΨ_

    Initial transformation estimate: $\left[R_{0},{\vec{t}}_{0}\right]$

    Upper bound on match uncertainty: $\sigma_{\text{max}}^{2}$       (default: ∞)

    Chi-square threshold value for outliers: $\chi_{\text{thresh}}^{2}$     (default: 7.81)

**output**: Final transformation $\left[R,\vec{t}\right]$ that aligns the shapes *X* and Ψ

**1** Initialize transformation: $\left[R,\vec{t}\right]\;\leftarrow \;\left[R_{0},{\vec{t}}_{0}\right]$

**2** Initialize noise model: *σ* ^2^ ← 0

**3** Compute initial correspondences (Equ. 8):

$\left[{\vec{y}}_{i},M_{\text{y}}{}_{i},{\text{M}}_{\text{S}}{}_{\text{y}i}]\leftarrow {\text{C}}_{\text{mlp}}({\vec{x}}_{i},\Psi ,I,I,R,\vec{t},\right)$

**4** Skip to Step 6

**5** Compute most-likely correspondences (Equ. 8):

$\left[{\vec{y}}_{i},M_{\text{y}}{}_{i},M_{\text{S}}{}_{\text{y}i}]\leftarrow {\text{C}}_{\text{mlp}}({\vec{x}}_{i},\Psi ,M_{\text{x}}{}_{i}+M_{\text{S}}{}_{\text{x}i}+\sigma^{2}I,M_{\Psi }+M_{\text{S}\Psi },R,\vec{t},\right)$

**6** Update the match-uncertainty noise-model term (Equ. 4):

$\sigma^{2}\leftarrow \min\left(\frac{1}{N_{\text{inlier}}}\sum_{i\;\in \;\text{inliers}}\|{\vec{y}}_{i}-R{\vec{x}}_{i}-\vec{t}\;{\|}_{2}^{2},\sigma_{\text{max}}^{2}\right)$

**7** Identify outliers using a chi-square test (Equ. 6):

$({\vec{x}}_{i},{\vec{y}}_{i}){\;\text{is}\;\text{outlier}\;\text{if}\;\text{E}}_{\text{SqrMahalDist}}({\vec{x}}_{i},{\vec{y}}_{i},M_{\text{x}i},M_{\text{y}i}+\sigma^{2}I,R,\vec{t})>\chi_{\text{thresh}}^{2}$

 and update the outlier noise-model terms (Equ. 7):

$\begin{array}{c} \varphi_{i}=\left\{\begin{array}{cc} 9∥{\vec{y}}_{i}-R{\vec{x}}_{i}-\vec{t}{\;∥}_{2}^{2} & \;\text{if}\;\left({\vec{x}}_{i},{\vec{y}}_{i}\right)\;\text{is}\;\text{an}\;\text{outlier}\\ 0 & \;\text{otherwise} \end{array}\right. \end{array}$

**8** Set the noise-model covariances for the registration phase:

$M_{\text{x}i}^{*}\leftarrow M_{\text{x}i}+M_{\text{Sx}i}+\frac{\varphi_{i}}{2}I$, $M_{\text{y}i}^{*}\leftarrow M_{\text{y}i}+M_{\text{Sy}i}+\frac{\varphi_{i}}{2}I+\sigma^{2}I$

**9** Update the transformation to align the corresponding point sets by GTLS (Equ. 20):

$\left[R,\vec{t}]\leftarrow \underset{\left[R,\vec{t}\right]}{\text{argmin}}\;\sum_{i=1}^{n}{\left({\vec{y}}_{i}-R{\vec{x}}_{i}-\vec{t}\right)}^{T}{\left(RM_{\text{x}i}^{*}R^{T}+M_{\text{y}i}^{*}\right)}^{-1}({\vec{y}}_{i}-R{\vec{x}}_{i}-\vec{t}\right)$

**10** **if** *not converged* **then** goto Step 5

The measurement-error noise models for the source point set and for a corresponding target point set are defined using two sets of covariance matrices *M*_X_ = {*M*_x*i*_} and *M*_Y_ = {*M*_y*i*_}, where *M*_Y_ is drawn from a larger set of covariances, *M*_Ψ_, that represents the entire target shape. *M*_Ψ_ may be either a superset of covariances or a rule for computing a covariance given any point on the target shape.

In addition to the covariances used to model measurement error, IMLP includes explicit support for a second set of noise-model covariances *M*_SX_ = {*M*_Sx*i*_} and *M*_SY_ = {*M*_Sy*i*_}, which are useful for modeling the locally-linear surface patches surrounding each point of a point-cloud shape model. These “surface-model” covariances are added to the measurement-error covariances to obtain the complete noise model for each point. The idea behind the surface-model covariances is to increase the noise-model variance in the surface-parallel directions in order to encourage match errors to be directed along the surface rather than perpendicular to the surface, thereby achieving closer alignment of the underlying surfaces being represented by the point-cloud shape models. This idea forms the basis of the GICP algorithm [11] and was also investigated in [12]. The IMLP algorithm treats the surface-model covariances separately from the measurement-error covariances in order to exclude the surface model from the outlier detection stage, which was found to improve the algorithm’s ability to reject outliers.

As indicated in the algorithm summary, IMLP’s noise model includes additional dynamically computed terms besides the input covariances. The “match-uncertainty” term (*σ* ^2^) attempts to account for uncertainty in the match process by adding an isotropic variance to the noise model with a magnitude equal to the estimated amount of misalignment between the source and target shapes. In the initial iterations of the algorithm, the residual error between corresponding points is largely due to shape misalignment; thus, the input covariances may not accurately represent the underlying distribution of match errors at first. As the algorithm iterates and the misalignment is reduced, the input covariances are expected to more accurately represent the distribution of match errors. To account for this effect, we follow a similar approach to Estépar et al. [10] and model the match uncertainty as an isotropic noise term having variance equal to the average square residual distance between the corresponding points. However, unlike [10], which includes all match errors in the estimate, we only include match errors from the current set of inliers when computing the match-uncertainty term

$$\begin{array}{c} \sigma^{2}=\frac{1}{N_{\text{inlier}}}\sum_{i\;\in \;\text{inlier}}{∥{\vec{y}}_{i}-R{\vec{x}}_{i}-\vec{t}\;∥}_{2}^{2} \end{array}$$ (4)

which has an intuitive appeal and which we found to improve IMLP’s performance with respect to outlier rejection. Note that *N*_inlier_ represents the number of matches forming the current set of inliers. A detailed justification of this model for estimating match uncertainty is addressed in [6].

Because the match-uncertainty term is isotropic, it may be added to the noise-model covariances of either the source or target points with the same effect. Since the match uncertainty intuitively affects the choice of correspondences, for the registration phase we choose to add this term to the covariances of the target points in Step 8 of Algorithm 2. However, for the correspondence search phase in Step 5, the match-uncertainty term is added to the covariances of the source points, rather than the target points, because this reduces computation in the correspondence phase. Note that because computing *σ* ^2^ requires having a set of correspondences in-hand, a fully isotropic noise model is used for the initialization of correspondences in Step 3.

The match-uncertainty term described above has importance for the chi-square outlier detection test in Step 7 of Algorithm 2. The match-uncertainty term enables the algorithm to converge robustly and quickly in the case of large initial misalignment by accounting for this misalignment in the noise model and preventing an overabundance of matches from being flagged as outliers based on the measurement-error covariances alone. In the case of registering a source and target shape having only partial overlap, it could happen that the average square match distance remains large even at the properly registered alignment. In this case, it may be desirable to prevent the match-uncertainty term from growing too large. To address this issue, we define a maximum threshold ($\sigma_{\text{max}}^{2}$) on the match uncertainty as an optional input to the IMLP algorithm. If no value is specified by the user, then the maximum threshold is disabled by setting it to a very large value.

Robustness to outliers is enabled via a chi-square test, which is used to identify outlier matches in Step 7 of Algorithm 2. Under an assumption of correspondence and of generalized Gaussian noise, the square Mahalanobis distance between matched points in 3D space is distributed as the sum of squares of three independent normalized Gaussian distributions, each representing a distribution along a different eigenvector of the noise-model covariance matrix. Thus, under the stated assumptions, the square Mahalanobis match distance has a chi-square distribution with three degrees of freedom [33]. Outliers are therefore detected by comparing each square Mahalanobis match distance

$$\begin{array}{c} {\text{E}}_{\text{SqrMahalDist}}\left(\vec{x},\vec{y},M_{\text{x}},M_{\text{y}},R,\vec{t}\;\right)={\left(\vec{y}-R\vec{x}-\vec{t}\;\right)}^{T}{\left(RM_{\text{x}}R^{T}+M_{\text{y}}\right)}^{-1}\left(\vec{y}-R\vec{x}-\vec{t}\;\right) \end{array}$$ (5)

to the value of the inverse cumulative density function (CDF) of a chi-square distribution with three degrees of freedom evaluated at some probability *p*. If a square Mahalanobis match distance exceeds this chi-square inverse CDF value ($\chi_{\text{thresh}}^{2}$) then that match is considered an outlier. Thus, a matched point-pair, $\left(\vec{x},\vec{y}\right)$, with corresponding noise covariances, *M*_x_ and *M*_y_, is an outlier if

$$\begin{array}{c} {\text{E}}_{\text{SqrMahalDist}}\left(\vec{x},\vec{y},M_{\text{x}},M_{\text{y}},R,\vec{t}\;\right)>chi2inv\left(p,3\right)=\chi_{\text{thresh}}^{2} \end{array}$$ (6)

where chi2inv(*p*, 3) is the chi-square inverse CDF function with three degrees of freedom evaluated at probability *p*. The chi-square inverse CDF threshold ($\chi_{\text{thresh}}^{2}$) is specified as an optional input parameter to the IMLP algorithm, which enables the user to adapt the algorithm to different percentages of outliers present in the shape data. Setting this threshold to a very large value effectively disables outlier detection. Disabling outlier detection in this manner may be useful in cases where the data is known to be free from outliers or possibly cases where a large initial misalignment is present, although the match-uncertainty term (*σ* ^2^) already functions as an automatic mechanism to account for large initial misalignment. When no chi-square inverse CDF threshold is specified by the user, the default threshold of 7.81 is used, which corresponds to a chi-square inverse CDF probability of *p* = 0.95.

To reduce the influence of outliers on the computed registration, a set of outlier noise-model terms ({*φ*_*i*_}) are used to bring additional isotropic variance into the noise models of the matches identified to be outliers. The effect of this added variance is to reduce the influence of the outliers in the registration phase [10], which occurs at Step 9 in Algorithm 2. If a match is determined to be an outlier then the outlier noise term, *φ*_*i*_, corresponding to that match is set equal to the square Euclidean distance between the matched points times some factor; otherwise, the outlier term is set to zero (7).

φi=9∥y⃗i-Rx⃗i-t⃗∥22if(x⃗i,y⃗i)isanoutlier0otherwise (7)

While we have used the factor 9 in our implementation (which brings the outlier match errors within approximately 1/3 standard deviation relative to their noise models), this factor could be reduced or increased to give respectively more or less weight to the outliers if desired.

Alternatively, to completely remove all outlier influence from the registration phase, any matches identified as outliers could be simply removed from the set of matches used to compute the registration in Step 9 of Algorithm 2. This strategy is preferred for cases such as registering shapes having only partial overlap, since the systematic tug from the large body of non-overlapping points could then be significant enough to affect the final accuracy of the registration. For small to moderate percentages of random outliers, our experience has been that inflating the variance works just as well as disregarding the matches entirely.

In our implementation of IMLP, we terminate the algorithm when the magnitudes of change in the transformation parameters *R* and $\vec{t}$ remain below some termination threshold values for two consecutive iterations or when a maximum number of iterations has been reached. For translation $\left(\vec{t}\right)$, the magnitude of change is simply computed as the norm of the change in the translation vector. For rotation (*R*), the Rodrigues form for the change in rotation is computed [34] and the angular component (the norm of the Rodrigues vector) is extracted as the magnitude of angular change. The threshold values used in the studies reported for this paper were 0.001 degrees rotation and 0.001 millimeters translation. We note that alternative termination criteria could also be used as substitute for our own.

Due to modifying the underlying noise models during each iteration, the IMLP algorithm cannot be guaranteed to converge. A similar scenario is encountered for many related ICP-based methods, with an in-depth discussion being found in [4]. Because of the possibility for non-convergence, we have added cycling detection as a further termination condition for IMLP. Cycling is detected by monitoring the value of the cost function being minimized within the registration phase. If the minimal cost computed by the registration phase increases twice within a period of four iterations and if the cost following the second increase is within a small tolerance of the cost following the first increase, then a cycle has been detected. In such cases, the algorithm terminates and returns the registration corresponding to the last iteration in which the cost function decreased. This termination condition is primarily a precaution to ensure computational efficiency, as a cycling condition would terminate at the maximum iteration count in any case.

Concerning IMLP’s ability to converge to the correct global solution, we note that, like other ICP-based methods, it is important to begin the registration close enough to the optimal solution in order to prevent converging to an incorrect solution created by local minima.

### IMLP Correspondence Phase: An Efficient PD-Tree Search Strategy for Computing Most-Likely Correspondences

In this subsection we describe our approach to efficiently compute the most-likely matches from the target shape, being those matches that maximize the match-likelihood function previously defined in (3) as indicated by the most-likely-point operator

$$\begin{array}{c} {\text{C}}_{\text{MLP}}\left(\vec{x},\Psi ,M_{\text{x}},M_{\Psi },R,\vec{t}\;\right)=\underset{\left[\vec{y},\;M_{\text{y}}\right]\;\in \;\left[\Psi ,\;M_{\Psi }\right]}{\text{argmax}}{\text{L}}_{\text{match}}\left(\vec{x},\vec{y},M_{\text{x}},M_{\text{y}},R,\vec{t}\;\right)\;. \end{array}$$ (8)

Maximizing the match-likelihood function of (3) simplifies to minimizing the “match-error function”

$$\begin{array}{cc} {\text{E}}_{\text{match}}(\vec{x},\vec{y},M_{\text{x}},M_{\text{y}},R,\vec{t}\;)=\\ & \log|RM_{\text{x}}R^{T}+M_{\text{y}}|+{\left(\vec{y}-R\vec{x}-\vec{t}\;\right)}^{T}{\left(RM_{\text{x}}R^{T}+M_{\text{y}}\right)}^{-1}\left(\vec{y}-R\vec{x}-\vec{t}\;\right)\;. \end{array}$$ (9)

It is important to not disregard the log term within the match-error function when computing the most-likely match, since the noise-model covariances may vary substantially over the target shape in general. Note that both the magnitude of the target covariances (i.e. the eigenvalues) and the orientation of the target covariances (i.e. the directions of the eigenvectors) may significantly alter the value of the log term. Thus, even if the covariance magnitude is fixed for all target noise models, the log term still has an impact for anisotropic distributions that have different orientations at different points on the target surface. If both the magnitudes and orientations of the noise-model covariances are constant across the entire target shape, then minimizing the match-error function reduces to that of minimizing the square Mahalanobis distance term in (9).

Algorithms 3 and 4 provide a summary of our efficient strategy for computing the most-likely correspondences. Note that in order to simplify the expressions in the summary we represent the noise-model of a target point by the single covariance *M*_y_. However, as previously noted, for the IMLP algorithm a target-point noise model is actually represented by two covariances, *M*_y_ and *M*_Sy_, in order to distinguish between components for the measurement error and for the local surface model. For the purposes of this subsection, no distinction between the noise-model components is required, and we will consider *M*_y_ to represent the total noise-model of a target point, i.e. (*M*_y_ + *M*_Sy_) as defined in the algorithm summary for IMLP. In an actual implementation having both types of noise-model components defined over the target shape, each type of covariance would be stored and returned separately along with the most-likely match.

**Algorithm 3.** PD-Tree Search for Most-Likely Correspondence

**input**: Source point: $\vec{x}$

   Source-point noise model: *M*_x_

   PD tree containing target shape (Ψ) and target noise model (*M*_Ψ_): *T*

   Current transformation: $\left[R,\vec{t}\right]$

   Prior most-likely match for this source point: $\left({\vec{y}}_{\text{pre}},M_{\text{y}\_\text{pre}}\right)$

**output**: Most-likely match and its corresponding noise model: $\left(\vec{y},M_{\text{y}}\right)$

**1** Initialize most-likely match to the prior match:

$\left[\vec{\text{y}},{\text{M}}_{\text{y}},E_{\text{best}}]\leftarrow [{\vec{y}}_{\text{prev}},M_{\text{y}\_\text{prev}},E_{\text{match}}(\vec{x},{\vec{y}}_{\text{prev}},M_{\text{x}},M_{\text{y}\_\text{prev}},R,\vec{t})\right]$

**2** Search for more-likely match in the left child of the PD-tree root node:

$\left[{\vec{\text{y}}}_{\text{LChild}},M_{\text{y}\_\text{LChild}},E_{\text{LChild}}]\leftarrow \text{NodeSearch}\;(T.Root.LChild,{\text{E}}_{\text{best}},\vec{x},M_{\text{x}},R,\vec{t}\right)$

**3** **if** *E*_*LChild*_ < *E*_*best*_ **then** update most-likely match:

$\left[\vec{y},M_{\text{y}},E_{\text{best}}\right]\leftarrow \left[{\vec{y}}_{\text{LChild}},M_{\text{y}\_\text{LChild}},E_{\text{LChild}}\right]$

**4** Search for more-likely match in the right child of the PD-tree root node:

$\left[{\vec{\text{y}}}_{\text{RChild}},M_{\text{y}\_\text{RChild}},E_{\text{RChild}}]\leftarrow \text{NodeSearch}\;(T.Root.RChild,{\text{E}}_{\text{best}},\vec{x},M_{\text{y}\_\text{RChild}},R,\vec{t}\right)$

**5** **if** *E*_*RChild*_ < *E*_*best*_ **then** update most-likely match:

$\left[\vec{y},\;M_{\text{y}},\;E_{\text{best}}]\;\leftarrow \;[{\vec{y}}_{\text{RChild}},\;M_{\text{y\_RChild}},\;E_{\text{RChild}}\right]$

Our search strategy for computing the most-likely correspondences is based on a modified PD tree formed around the target shape. The distinguishing element of a PD-tree data structure, in comparison to the standard KD-tree data structure, is that each node of the tree has its own unconstrained local coordinate system rather than requiring the local coordinate frame of each node to be axis-aligned with a common global coordinate system.

A node of the PD-tree is constructed by first computing the covariance of the positions of all datums assigned to the node. A datum is defined to be one of the smallest principle elements comprising the target shape, such as a point from a point cloud or a triangle from a mesh. After the covariance of datum positions is computed, the local coordinate system of the node is defined by aligning the coordinate axes of the node with the eigenvectors of the covariance matrix and positioning the node origin at the mean datum position. It is customary to align the x-axis along the direction of greatest variance (i.e. along the eigenvector associated with the largest eigenvalue). Finally, a bounding box of minimal size is constructed that is axis-aligned with the local coordinate system of the node and that fully contains all datums within the node. The node is then split along the local x-axis (along the direction of greatest variance) in order to form the left and right child nodes, and the process continues down the tree until either the number of datums within the node or the size of the node’s bounding box falls below a threshold value. To begin this process, the root node of the PD tree is formed by assigning to it all datums comprising the entire target shape.

**Algorithm 4.** NodeSearch Function for the PD-Tree Search

**input**: Node of PD tree being search: 𝓝

   Source point: $\vec{x}$

   Source-point noise model: *M*_*x*_

   Current transformation: $[R,\;\vec{t]}$

   Current best match error: *E*_best_

**output**: Best match within node: ${\vec{y}}_{\text{node}}$

   Noise model of best match within node: *M*_y_node_

   Updated best match error: *E*_node_

**1** Initialize the best match within this node:

$\left[E_{\text{node}},\;{\vec{y}}_{\text{node}},\;M_{\text{y\_node}}]\;\leftarrow \;[E_{\text{best}},\;0,\;0\right]$

**2** Compute an ellipsoid bound (𝓔) centered at the transformed source point $\left(R\vec{x}\;+\;\vec{t}\right)$ within which candidates for a better match may be found:

$𝓔\;=\;\{\vec{z}\;|\;{\left(\vec{z}\;-\;R\vec{x}\;-\;\vec{t}\right)}^{T}\;{\left(M_{\text{sub}}\right)}^{-1}\;(\vec{z}\;-\;R\vec{x}\;-\;\vec{t})\;\leq \;E_{\text{best}}\;-\;log_{\text{min}}\}$

   See Equations (13) and (14, 15, or 17)

**3 if** 𝓔 *intersects* 𝓝.*OBB* **then**

**4** **if** 𝒩 *is a leaf node* **then**

**5** **foreach** *datum*_*i*_ ∈ 𝒩 **do**

**6**      Compute the most-likely match on *datum*_*i*_ to get:

$\left[{\vec{y}}_{\text{datum}},\;M_{\text{y\_datum}},\;E_{\text{datum}}\right]$ (S1 Appendix)

**7** **if** *E*_datum_ < *E*_node_ **then** update best match in node:

$\left[{\vec{y}}_{node},\;M_{\text{y\_node}},\;E_{\text{node}}]\;\leftarrow \;[{\vec{y}}_{\text{datum}},\;M_{\text{y\_datum}},\;E_{\text{datum}}\right]$

**8** **end**

**9** **else**

**10**    Search left child node:

$\left[{\vec{y}}_{\text{LChild}},\;M_{\text{y\_LChild}},\;E_{\text{LChild}}]\;\leftarrow \;\text{NodeSearch}\;\text{(}𝓝.LChild,E_{\text{node}},\;\vec{x},\;M_{\text{x}},\;R,\;\vec{t}\right)$

**11** **if** *E*_LChild_ < *E*_node_ **then** update the most-likely match for this node:

$\left[{\vec{y}}_{\text{node}},\;M_{\text{y\_node}},\;E_{\text{node}}]\;\leftarrow \;[{\vec{y}}_{\text{LChild}},\;M_{\text{y\_LChild}},\;E_{\text{LChild}}\right]$

**12**    Search right child node:

$\left[{\vec{y}}_{\text{RChild}},\;M_{\text{y\_RChild}},\;E_{\text{RChild}}]\;\leftarrow \;\text{NodeSearch}\;\text{(}𝓝.RChild,E_{\text{node}},\;\vec{x},\;M_{\text{x}},\;R,\;\vec{t}\right)$

**13** **if** *E*_RChild_ < *E*_node_ **then** update most-likely match for this node:

$\left[{\vec{y}}_{\text{node}},\;M_{\text{y\_node}},\;E_{\text{node}}]\;\leftarrow \;[{\vec{y}}_{\text{RChild}},\;M_{\text{y\_RChild}},\;E_{\text{RChild}}\right]$

**14** **end**

**15 end**

 Node Object Parameters:

  Datums and corresponding noise-model covariances in this node: {*datum*_*i*_, *M*_y*i*_}

  Oriented bounding box bounding all datums in this node: *OBB*

  Node noise model used to form a lower bound on match errors within this node:

    {*λ*_node_min, *i*_} and either *M*_node_ or *λ*_node_max_

    (depends on the bounding method chosen in Step 2 of NodeSearch)

To illustrate the process of computing a most-likely match, suppose that we are given a source point $\vec{x}$ having noise covariance *M*_x_ and that we are given a current candidate for the most-likely match on the target shape having match error *E*_best_. The search for the most-likely correspondence begins at the root node of the PD tree and progressively makes it way down the tree until reaching the leaf nodes. Whenever a leaf node is encountered then match errors are computed for every datum within the leaf node and the current candidate for most-likely match is updated whenever a match error smaller than *E*_best_ is found. When the search that began at the root node is complete then the final candidate for most-likely match will be the most-likely match.

In order to perform the PD-tree search efficiently, the problem to consider before searching deeper within a given node is whether or not it is possible for a target point located anywhere within the node boundary to produce a match error that is lower than the current best match error, *E*_best_. If a lower match error is not possible within the bounds of the node, then that node and all nodes below it may be safely skipped. Consider testing a match for which all inputs of the match-error equation (9) are known, except the position of the target point $\vec{y}$. Our goal then is to determine whether any point located within the node bounds can produce a match error less than *E*_best_. Introducing this inequality into (9) and shifting the log term to the opposite side defines the equation of an ellipsoid centered at the position of the transformed source point $\left(R\vec{x}\;+\;\vec{t}\right)$ as given by

$$\begin{array}{c} {\left(\vec{y}-R\vec{x}-\vec{t}\;\right)}^{T}{\left(RM_{\text{x}}R^{T}+M_{\text{y}}\right)}^{-1}\left(\vec{y}-R\vec{x}-\vec{t}\;\right)<E_{\text{best}}-\log|RM_{\text{x}}R^{T}+M_{\text{y}}|\;. \end{array}$$ (10)

Any target point $\vec{y}$ that produces a match error lower than *E*_best_ will be located within this ellipsoid boundary. The task now is to determine whether the ellipsoid so defined intersects the oriented bounding box (OBB) of the node. If intersection exists, then it is possible that the node may contain a better match. If intersection does not exist, then the given node and all nodes below it cannot contain a better match and may be skipped in the continuing search. To compute the ellipsoid-OBB intersection test, we employ the efficient method described in [35].

The problem of bounding the match error of a node is actually more complicated than indicated above, because different points on the target shape may have different noise-model covariances. Thus, the covariance *M*_y_ is not static within a node. To address this issue, a substitute (*M*_sub_) for the match covariance (*RM*_x_ *R* ^*T*^ + *M*_y_) is required that produces a lower bound on the match error for any target point within the node relative to the match error obtained when using the target point’s true noise-model covariance. In other words, the ellipsoid bound that results from applying the substitute covariance *M*_sub_ in (10) must fully contain all ellipsoid bounds that result from using any covariance in the set {(*RM*_x_ *R* ^*T*^ + *M*_y*i*_)} for all {*M*_y*i*_} represented within the node. Note that the covariance expression (*RM*_x_ *R* ^*T*^ + *M*_y_) appears twice in (10), within both a log and a square Mahalanobis-distance term. In the discussion that follows, we will consider independent replacements for the covariance expressions within each term.

#### Log-Component Bound

For the log term in (10) we seek a lower bound from the set of covariances represented within the node, since smaller log values increase the size of the ellipsoid boundary. Consider the two covariances *RM*_x_ *R* ^*T*^ and *M*_y_ each having known eigenvalues {*λ*_x,1_, *λ*_x,2_, *λ*_x,3_} and {*λ*_y,1_, *λ*_y,2_, *λ*_y,3_}, respectively, being arranged in order of increasing magnitude. A lower bound on the determinant of the sum of the two covariances is then given by

$$\begin{array}{c} |RM_{\text{x}}R^{T}+M_{\text{y}i}|\geq \prod_{i=1}^{3}\left(\lambda_{\text{x},i}+\lambda_{\text{y},i}\right) \end{array}$$ (11)

as proven in [36]. It is clear from the eigen decompositions of the sum of covariances

$$\begin{array}{c} RM_{\text{x}}R^{T}+M_{\text{y}}=RV_{\text{x}}\mathrm{diag}\left(\lambda_{\text{x},1},\lambda_{\text{x},2},\lambda_{\text{x},3}\right)V_{\text{x}}^{T}R^{T}+V_{\text{y}}\mathrm{diag}\left(\lambda_{\text{y},1},\lambda_{\text{y},2},\lambda_{\text{y},3}\right)V_{\text{y}}^{T} \end{array}$$ (12)

where diag(*d*_1_, …, *d*_*n*_) represents a diagonal matrix with the listed diagonal elements beginning from the upper left-hand corner, that this lower bound is achieved when the eigenvectors of *RM*_x_ *R* ^*T*^ are in alignment with the eigenvectors of *M*_y_ associated by eigenvalue rank, i.e. when *RV*_x_ = *V*_y_. A lower bound on the log term for an entire node is therefore made possible by computing the smallest eigenvalue within each rank order (i.e. each magnitude ordering) among all covariance matrices {*M*_y*i*_} represented in the node. The log term of (10) may then be replaced by

$$\begin{array}{c} log_{\text{min}}=\log\prod_{i=1}^{3}\left(\lambda_{\text{x},i}+\lambda_{\text{node\_min},i}\right) \end{array}$$ (13)

where *λ*_x, *i*_ are the eigenvalues of *Mx* by rank order and *λ*_node_min, *i*_ are the smallest eigenvalues within each rank order among all covariances {*Myi*} represented within the node. For example, *λ*_node_min,2_, corresponding to the second rank order, is computed by selecting the smallest value from the set of all second-rank eigenvalues represented within the node.

Note that in order to implement the log bound, eigen decompositions for the source and target noise-model covariances need be computed (or provided) only once, since other noise-model components added by the IMLP algorithm are isotropic and uniformly increase each eigenvalue, which does not require a new eigen decomposition. As an optimization, nodes may use the same *λ*_node_min, *i*_ values as used by their parent node whenever these values remain within some factor of the parent’s values. This enables the bound on the log term to be recomputed only when doing so significantly affects the ellipsoid boundary size rather than recomputing the log bound at every node visited.

#### Mahalanobis-Component Bound Method 1: Spherical Bound

In this and the two following sub-subsections we address the problem of determining a substitute covariance for bounding the square Mahalanobis-distance term in (10) for a given node. For the square Mahalanobis-distance term, we seek a replacement for the match covariance (*RM*_x_ *R* ^*T*^ + *M*_y_) that has a variance at least as large in any direction as that of any covariance from the set {(*RM*_x_ *R* ^*T*^ + *M*_y*i*_)} for all {*M*_y*i*_} represented within the node, since increasing the variance in some direction increases the size of the ellipsoid bound in that direction.

The first method that we describe for bounding the square Mahalanobis-distance term provides the simplest and least compact bound. The idea is to replace the entire match covariance (*RM*_x_ *R* ^*T*^ + *M*_y_) by the expression (*λ*_x_max_ + *λ*_node_max_)*I* where *λ*_x_max_ is the largest eigenvalue of the source-point covariance *M*_x_ and *λ*_node_max_ is the largest eigenvalue among all target-point covariances {*M*_y*i*_} represented within the node. Performing this substitution along with the substitution of the log bound simplifies (10) to

$$\begin{array}{c} {\left(\vec{y}-R\vec{x}-\vec{t}\;\right)}^{T}{\left(\lambda_{\text{x\_max}}+\lambda_{\text{node\_max}}\right)}^{-1}I\left(\vec{y}-R\vec{x}-\vec{t}\;\right)<E_{\text{best}}-log_{\text{min}}\;. \end{array}$$ (14)

The advantage of this method is that the bounding ellipsoid simplifies to a bounding sphere, which results in a sphere-OBB intersection test with the node, which is simpler and more efficient to compute. The simplicity of this method is offset, however, by the cost associated with forming a less compact bound, since a higher number of node searches are performed as a result.

#### Mahalanobis-Component Bound Method 2: Simple Ellipsoidal Bound

An improvement over the first method for bounding the square Mahalanobis-distance term in (10) may be achieved by finding a replacement for only *M*_y_ within the match covariance expression (*RM*_x_ *R* ^*T*^+*M*_y_). In this case, *M*_y_ is replaced by *λ*_node_max_ *I* where *λ*_node_max_ is as defined for the first bounding method. Performing this substitution and substituting for the log bound simplifies (10) to

$$\begin{array}{c} {\left(\vec{y}-R\vec{x}-\vec{t}\;\right)}^{T}{\left(RM_{\text{x}}R^{T}+\lambda_{\text{node\_max}}I\right)}^{-1}\left(\vec{y}-R\vec{x}-\vec{t}\;\right)<E_{\text{best}}-log_{\text{min}}\;. \end{array}$$ (15)

This method produces a bounding ellipsoid that is more compact than the bounding sphere of the prior method, yet remains simple to compute. As done for the log bound, a node may re-use the *λ*_node_max_ value of a parent node whenever its value remains within some factor of the parent’s value. This enables the ellipsoid bound to be recomputed only when doing so results in a significant reduction of the ellipsoid boundary rather than recomputing the covariance expression at every node visited.

#### Mahalanobis-Component Bound Method 3: Compact Ellipsoidal Bound

This final method for bounding the square Mahalanobis-distance term in (10) is the most compact but also the most complex bound. As previously mentioned, consider that a substitute for the match covariance must produce a bounding ellipsoid that fully contains all ellipsoid bounds that result when using any covariance from the set {(*RM*_x_ *R* ^*T*^+*M*_y*i*_)} for all {*M*_y*i*_} represented within the node. Further, consider that increasing the variance of *M*_y_ in any direction strictly increases the ellipsoid boundary defined by (10). The strategy then is to compute a new covariance that has a variance at least as large in all directions as any covariance {*M*_y*i*_} represented within the node while producing a bounding ellipsoid that is as compact as possible. This may be accomplished by computing a new covariance, *M*_node_, that defines the ellipsoid of minimal volume that fully contains the union of all ellipsoids produced by each covariance {*M*_y*i*_} represented within the node.

$$\begin{array}{ccc} M_{\text{node}} & = & \underset{M}{\text{argmin}}|M^{-1}|\;\text{such}\;\text{that}\;\text{the}\;\text{ellipsoid}\;\text{defined}\;\text{by}\;\left\{\vec{y}\;\in \;R^{3}\;|\;\vec{y}{\;}^{T}M^{-1}\vec{y}\leq 1\right\}\\ & & \;\text{fully}\;\text{contains}\;\text{the}\;\text{union}\;\text{of}\;\text{ellipsoids}\;\underset{i\;\in \;\text{node}}{⋃}\left\{\vec{y}\;\in \;R^{3}\;|\;\vec{y}{\;}^{T}M_{\text{y}i}^{-1}\vec{y}\leq 1\right\} \end{array}$$ (16)

Note that the covariance, *M*, computed in (16) is constrained to be a symmetric, positive-definite matrix. A method for approximating the minimal volume bounding ellipsoid of ellipsoids is addressed in [37]. Also note that *M*_node_ is computed only once for each node when constructing the PD tree and is thereafter stored as a property of the node. Performing the substitutions for *M*_node_ and for the log bound modifies (10) to be

$$\begin{array}{c} {\left(\vec{y}-R\vec{x}-\vec{t}\;\right)}^{T}{\left(RM_{\text{x}}R^{T}+M_{\text{node}}\right)}^{-1}\left(\vec{y}-R\vec{x}-\vec{t}\;\right)<E_{\text{best}}-log_{\text{min}}\;. \end{array}$$ (17)

As before, the covariance expression should be recomputed only when doing so substantially reduces the size of the ellipsoid boundary. Significant reduction of the ellipsoid bound may be determined by comparing the determinant of the node’s *M*_node_ to the determinant of its parent’s *M*_node_. If the node’s determinant is within some factor of the parent’s determinant, then the node may continue to use the same match covariance as its parent.

### IMLP Registration Phase: Aligning Corresponding Point Sets by Generalized Total Least Squares

In this section, we present our approach for computing the rigid-body alignment of two corresponding point sets such that the total match likelihood is maximized as defined by

$$\begin{array}{c} L_{\text{total}}\left(X,Y,M_{\text{X}},M_{\text{Y}}\right)=\max_{\left[R,\vec{t}\;\right]}\prod_{i=1}^{n}{\text{L}}_{\text{match}}\left({\vec{x}}_{i},{\vec{y}}_{i},M_{\text{x}i},M_{\text{y}i}\right) \end{array}$$ (18)

where the match-likelihood function for a single match (L_match_) was previously defined in (3). Maximizing the total match likelihood is equivalent to minimizing the total match error defined as

$$\begin{array}{c} \sum_{i=1}^{n}\log|RM_{\text{x}i}R^{T}+M_{\text{y}i}|+\sum_{i=1}^{n}{\left({\vec{y}}_{i}-R{\vec{x}}_{i}-\vec{t}\;\right)}^{T}{\left(RM_{\text{x}i}R^{T}+M_{\text{y}i}\right)}^{-1}\left({\vec{y}}_{i}-R{\vec{x}}_{i}-\vec{t}\;\right)\;. \end{array}$$ (19)

Unlike in the correspondence phase, the covariance matrices of the target points {*M*_y*i*_} are now fixed. Although the value of the log term may still be affected by a change in rotation, the impact of rotation on the square Mahalanobis-distance term is far more pronounced. Thus, we may disregard the log term in this phase, which simplifies the optimization considerably to that of minimizing a sum of square Mahalanobis distances

$$\begin{array}{c} {\text{E}}_{\text{GTLS}}\left(X,Y,M_{\text{X}},M_{\text{Y}}\right)=\min_{\left[R,\vec{t}\;\right]}\sum_{i=1}^{n}{\left({\vec{y}}_{i}-R{\vec{x}}_{i}-\vec{t}\;\right)}^{T}{\left(RM_{\text{x}i}R^{T}+M_{\text{y}i}\right)}^{-1}\left({\vec{y}}_{i}-R{\vec{x}}_{i}-\vec{t}\;\right) \end{array}$$ (20)

which has the form of a nonlinear generalized total-least-squares problem. This optimization is the same GTLS problem for registering corresponding point sets as found in prior ICP-based algorithms involving anisotropic noise models [10–12]. As mentioned in the introduction, methods of solution for this type of problem follow an iterative approach.

To derive our approach, we first express the problem in alternate form. It can be shown (S2 Appendix) that the unconstrained optimization of (20) is equivalent to the following constrained optimization

$$\begin{array}{ccc} {\text{E}}_{\text{GTLS}}(X,Y,M_{\text{X}},M_{\text{Y}}) & = & \min_{\left[R,\vec{t}\;\right]}\sum_{i=1}^{n}{\left({\vec{x}}_{i}-{\vec{x}}_{i}^{\;*}\right)}^{T}M_{\text{x}i}^{-1}\left({\vec{x}}_{i}-{\vec{x}}_{i}^{\;*}\right)+\sum_{i=1}^{n}{\left({\vec{y}}_{i}-{\vec{y}}_{i}^{\;*}\right)}^{T}M_{\text{y}i}^{-1}\left({\vec{y}}_{i}-{\vec{y}}_{i}^{\;*}\right)\\ & & \text{subject}\;\text{to:}\;{\vec{y}}_{i}^{\;*}=R{\vec{x}}_{i}^{\;*}+\vec{t} \end{array}$$ (21)

where $\left\{{\vec{x}}_{i}^{\;*}\right\}$ and $\left\{{\vec{y}}_{i}^{\;*}\right\}$ represent the optimizer’s estimates of the unknown, noise-free positions of the source and target point-pairs, which, due to the correspondence assumption, are constrained to have perfect alignment. Thus, our goal is to solve the transformation parameters, *R* and $\vec{t}$, that minimize (21) subject to a perfect alignment constraint on the unknown, noise-free point positions. The derivation of our strategy was particularly aided by the works of [38, 39] regarding the topic of total-least-squares estimation.

The first step in the derivation is to re-express the constraints of (21) as

$$\begin{array}{c} {\text{F}}_{i}\left({\vec{x}}_{i}^{\;*},{\vec{y}}_{i}^{\;*},R,\vec{t}\;\right)={\vec{y}}_{i}^{\;*}-R{\vec{x}}_{i}^{\;*}-\vec{t}=0 \end{array}$$ (22)

and then to linearize these constraints using a first-order Taylor-series expansion centered at the known values ${\vec{y}}_{i}$, ${\vec{x}}_{i}$, *R*_0_, and ${\vec{t}}_{0}$, where *R*_0_ and ${\vec{t}}_{0}$ are initial estimates of the transformation. Note that we define *R*_*k*_ and ${\vec{t}}_{k}$ to be the estimates of the transformation parameters that are computed at each iteration *k*. Performing a linearization of the rotation matrix leads to the skew approximation form for an incremental rotation as defined by

$$\begin{array}{c} \Delta R\approx I+\mathrm{skew}(\Delta \vec{\alpha }) \end{array}$$ (23)

$$\begin{array}{c} \mathrm{skew}\left(\Delta \vec{\alpha }\right)=\left[\begin{array}{ccc} 0 & -\Delta \alpha_{\text{z}} & \Delta \alpha_{\text{y}}\\ \Delta \alpha_{\text{z}} & 0 & -\Delta \alpha_{\text{x}}\\ -\Delta \alpha_{\text{y}} & \Delta \alpha_{\text{x}} & 0 \end{array}\right]\;. \end{array}$$ (24)

This parameterization enables representing small-angle rotations as a 3D vector, $\Delta \vec{\alpha }\;=\;{\left[\Delta \alpha_{\text{x}},\;\Delta \alpha_{\text{y}},\;\Delta \alpha_{\text{z}}\right]}^{T}$. We also note that using Lie algebra and exponential maps to parameterization the rotation, rather than the skew-approximation form described here, may also be a very effective approach for solving this problem. Note that skew$(\vec{x})\vec{y}$ is simply matrix notation for the cross product $\left(\vec{x}\;\times \;\vec{y}\right)$; thus, the positions of $\vec{x}$ and $\vec{y}$ may be interchanged by negation, which is implicitly used in forming the Taylor-series expansion of the constraint equations below. Using $\Delta \vec{\alpha }$ to represent change in rotation and defining $\Delta \vec{t}$ to be change in translation, with some algebraic manipulation the constraints of (22) may be linearized to the approximate form

$$\begin{array}{c} {\text{F}}_{\text{L}i}^{k}\left({\vec{x}}_{i},{\vec{y}}_{i},\Delta \vec{\alpha },\Delta \vec{t}\;\right)={\text{F}}_{i}^{\text{0}}\left({\vec{x}}_{i},{\vec{y}}_{i},R_{k},{\vec{t}}_{k}\right)-r_{\text{y}i}+R_{k}r_{\text{x}i}+\mathrm{skew}\left(R_{k}{\vec{x}}_{i}\right)\Delta \vec{\alpha }-\Delta \vec{t}=0 \end{array}$$ (25)

which is linear with respect to change in rotation $\Delta \vec{\alpha }$ and change in translation $\Delta \vec{t}$. Here we define ${\text{F}}_{i}^{0}({\vec{x}}_{i},{\vec{y}}_{i},R_{k},{\vec{t}}_{k})={\vec{y}}_{i}-R_{k}{\vec{x}}_{i}-{\vec{t}}_{k}$, $r_{\text{y}i}={\vec{y}}_{i}-{\vec{y}}_{i}^{\;*}$, $r_{\text{x}i}={\vec{x}}_{i}-{\vec{x}}_{i}^{\;*}$, $R_{k+1}=\;(\Delta R)R_{k}\;\approx \;(I\;+\;\text{skew(}\Delta \vec{\alpha }))R_{k}$, and ${\vec{t}}_{k+1}\;=\;{\vec{t}}_{k}\;+\;\Delta \vec{t}$, where *k* denotes an iteration of the optimization procedure.

The next step in the derivation is to apply the method of Lagrange multipliers to enforce the linearized constraints while minimizing the cost function. The Lagrange function becomes

$$\begin{array}{c} 𝓛\left(\Delta \vec{\alpha },\Delta \vec{t},\lambda \right)=\sum_{i=1}^{n}r_{\text{x}i}^{T}M_{\text{x}i}^{-1}r_{\text{x}i}+\sum_{i=1}^{n}r_{\text{y}i}^{T}M_{\text{y}i}^{-1}r_{\text{y}i}+\sum_{i=1}^{n}\lambda_{i}^{T}{\text{F}}_{\text{L}i}^{k}\left({\vec{x}}_{i},{\vec{y}}_{i},\Delta \vec{\alpha },\Delta \vec{t}\;\right) \end{array}$$ (26)

where *λ* = {*λ*_*i*_} represents the set of Lagrange multipliers with each *λ*_*i*_ being a 3-vector. Next is solving for the zero derivatives of the Lagrange function with respect to the residuals {*r*_x*i*_} and {*r*_y*i*_}, the change in transformation parameters $\Delta \vec{\alpha }$ and $\Delta \vec{t}$, and the Lagrange multipliers. After making substitutions between these differential equations, we finally obtain (27) for computing an incremental update of the transformation parameters $\Delta p\;=\;{\left[\Delta \vec{\alpha },\;\Delta \vec{t}\right]}^{T}$.

$$\begin{array}{c} J^{T}M^{-1}J\Delta p=-J^{T}M^{-1}F^{0}\\ \Delta p=\left[\begin{array}{c} \Delta \vec{\alpha }\\ \Delta \vec{t} \end{array}\right]\;F^{0}=\left[\begin{array}{c} F_{1}^{0}\\ \vdots \\ F_{n}^{0} \end{array}\right]\;J=\left[\begin{array}{cc} \mathrm{skew}(R_{k}{\vec{x}}_{1}) & -I\\ \vdots & \vdots \\ \mathrm{skew}(R_{k}{\vec{x}}_{n}) & -I \end{array}\right]\;M=\left[\begin{array}{c} F_{\text{x}}^{0}M_{\text{x}}{F_{\text{x}}^{0}}^{T}+M_{\text{y}} \end{array}\right]\\ F_{\text{x}}^{0}=\left[\begin{array}{ccc} -R_{k} & & \\ & \ddots & \\ & & -R_{k} \end{array}\right]\;M_{\text{x}}=\left[\begin{array}{ccc} M_{\text{x}1} & & \\ & \ddots & \\ & & M_{\text{x}n} \end{array}\right]\;M_{\text{y}}=\left[\begin{array}{ccc} M_{\text{y}1} & & \\ & \ddots & \\ & & M_{\text{y}n} \end{array}\right] \end{array}$$ (27)

Here *F* ^0^ is defined as a stacked vector of match residuals, *J* is the Jacobian matrix of the constraints relative to the transformation parameters, and *M* is the complete covariance matrix for all matches. Since the match errors are assumed to be independent between matches, *M* has a 3 x 3 symmetric, positive-definite, block-diagonal structure for points in 3D. Simplifying this solution for registrations in 2D is trivial.

The resulting expression in (27) is a linear system of six equations having the recognizable form *Ax* = *b*, where *A* is 6 x 6 symmetric. This expression is further recognized to have the form of an update equation from the nonlinear Gauss-Newton method of nonlinear optimization. The solution to (21) is computed by iteratively solving (27) using standard least squares, with each solution providing an incremental update (Δ*p*) for the current transformation parameter estimates *R*_*k*_ and ${\vec{t}}_{k}$, which are updated as

$$\begin{array}{c} R_{k+1}=R\left(\Delta \vec{\alpha }\right)R_{k}\;,\;{\vec{t}}_{k+1}={\vec{t}}_{k}+\Delta \vec{t} \end{array}$$ (28)

where $R(\Delta \vec{\alpha })$ is as defined in the following paragraph. The linear system of (27) is re-solved following each update until the transformation estimates converge. This approach is nearly equivalent to the standard Gauss-Newton method, with a modification being that the covariance matrices are updated at each iteration.

In (28) we define $R(\Delta \vec{\alpha })$ to be a rotation matrix computed using the Rodrigues rotation formula [34] about an axis oriented along $\Delta \vec{\alpha }$ and having a rotation angle of $\left||\Delta \vec{\alpha }|\right|$ radians. Using the Rodrigues form $R(\Delta \vec{\alpha })$ rather than the skew approximation form $\left(I\;+\;\text{skew(}\Delta \vec{\alpha })\right)$ ensures that *R*_*k*+1_ always satisfies the conditions for being a valid rotation matrix.

Since the linear system of (27) is symmetric, an efficient and stable approach for solving the least-squares iterates is to use Cholesky or *LDL* ^*T*^ decomposition. In our implementation we employ the more general SVD decomposition, since the symmetric decompositions were not supported by the numerical libraries used in our implementation. Because the linear system is small, SVD also provides reasonable efficiency. Note that, in the interest of efficiency, it is important to take advantage of the sparse structure of *M* when computing the matrix operations required to form this linear system.

We define the termination condition to be when the magnitude of incremental change in the transformation parameters fall below threshold values. In our implementation, we use convergence thresholds of 0.001 mm translation and 0.001 degrees rotation, but this may be defined by the user.

A summary of the approach described above for solving the GTLS problem of aligning two corresponding point sets is provided below as Algorithm 5.

**Algorithm 5.** GTLS Registration of Corresponding Point Sets

**input**: Corresponding source and target point sets: $X=\{{\vec{x}}_{i}\},Y=\{{\vec{y}}_{i}\}$

    Noise-model covariances for the source and target points:

*M*_x_ = {*M*_x*i*_, *M*_Y_ = {*M*_y*i*_}

    Initial transformation estimate: $\left[R_{0},{\vec{t}}_{0}\right]$

**output**: Final transformation that aligns the corresponding point sets: $\left[R,\vec{t}\right]$

**1** Initialize the transformation: $\left[R_{k},{\vec{t}}_{k}]\leftarrow [R_{0},{\vec{t}}_{0}\right]$

**2** Compute *F* ^0^ using *X, Y, R*_*k*_, and ${\vec{t}}_{k}$

**3** Compute *J* using *R*_*k*_ and *X*

**4** Solve incremental transformation $\Delta p={\left[\Delta \vec{\alpha },\Delta \vec{t}\right]}^{T}$ by standard least squares (27)

**5** Update the transformation parameters: $\left[R_{k},{\vec{t}}_{k}]\leftarrow [\text{R}(\Delta \vec{\alpha })R_{k},{\vec{t}}_{k}+\Delta \vec{t}\right]$

**6** **if** $\|\Delta \vec{\alpha }\|\leq \Delta \alpha_{\text{threshold}}\mathrm{and}\|\Delta \vec{t}\|\leq \Delta t_{threshold}$ **then**

**7**  Goto Step 2

**8 end**

**9** Return the final transformation: $\left[R,\vec{t}]\leftarrow [R_{k},{\vec{t}}_{k}\right]$

## Results and Discussion

In this section, we present our experimental results. We compare the IMLP algorithm to several competing methods under a wide range of test conditions including various isotropic and anisotropic noise levels, with and without outliers, and using different (i.e. mesh and point cloud) representations of various target shapes. Other methods evaluated for comparison with IMLP include standard ICP [1], a robust variant of ICP [4] (which we refer to as “Robust ICP”), GICP [11], and CPD [20]. For the non-outlier cases, near comparison is also made with GTLS-ICP [10] and A-ICP [12] using variants of our own method, IMLP-CP and IMLP-MD, respectively.

The two variants on IMLP directly compare the most-likely match criterion of IMLP with the closest-point (CP) match criterion used by GTLS-ICP and the Mahalanobis-distance (MD) match criterion used by A-ICP. Since only the matching phase of IMLP-CP and IMLP-MD has been modified with respect to IMLP, this comparison directly evaluates the merit of the three criterion for computing matches: closest-point matching (GTLS-ICP, IMLP-CP), Mahalanobis-distance matching (A-ICP, IMLP-MD), and most-likely-point matching (IMLP).

GICP and CPD appear in the experiments involving a point-cloud target shape and not in the experiments involving a mesh target shape. This is because CPD is limited by design to point-cloud-to-point-cloud registration, and GICP as well is most suited to the context of registering non-continuous representations of two surfaces (i.e. point clouds).

For the GICP and CPD algorithms, we have used the implementations made publicly available by their respective authors. For the remaining algorithms (standard ICP, Robust ICP, IMLP, IMLP-CP, IMLP-MD) our own implementations have been used. Minor changes were made to the source code of GICP and CPD in order to use the same termination criterion across all compared methods and, in the case of GICP, to orient the surface-model covariances directly along the known surface normal at each point rather than estimating the surface normals from neighboring points in the point-cloud. These various implementations are based on single-threaded programming in C++. Thus, all methods were evaluated on level ground in terms of the efficiency of the runtime environment, with an exception being that the CPD algorithm ran multi-threaded under certain settings (discussed later in the results). As a further minor caveat, the CPD implementation uses Matlab as a front-end while incorporating a C-compiled mex library for the heavy-lifting.

All registration methods were configured to terminate when the magnitude of change in the transformation parameters remain below threshold levels for two consecutive iterations or when a maximum iteration count is reached. The transformation thresholds were set to 0.001 mm translation and 0.001 degrees rotation with a maximum iteration count of 100 iterations (except where noted in the results). An advantage of using transformation thresholds as the basis for termination is that the need to normalize across the various cost functions employed by each algorithm is completely averted.

The algorithms that we have programmed (standard ICP, Robust ICP, IMLP, IMLP-CP, IMLP-MD) use the CISST [40] and WildMagic5 [41] C++ libraries for numerical linear algebra and standard least-squares computations. WildMagic5 is used for its efficient, non-iterative method of computing the eigen decomposition of a 3 x 3, symmetric, positive-definite matrix.

Before presenting a comparison of the algorithms described above, we begin the results section by evaluating our approach to solving the GTLS problem of registering two corresponding point sets. The proposed Gauss-Newton-based approach is compared to the prior methods of Estepar et al. [10] and Balachandran and Fitzpatrick [31], which have also been proposed to solve this specific problem. However, one limitation of the method as described by Balachandran and Fitzpatrick is that anisotropic noise is limited to the local coordinates of only one point set. All three methods share a commonality of being easy to program using a basic linear algebra library supporting a standard least-squares solver.

### Experiment 1: Generalized Total-Least-Squares Methods for Registering Corresponding Point Sets

In this study, we evaluate the proposed Gauss-Newton-based approach for computing the optimal rigid-body alignment that registers two corresponding point sets under anisotropic measurement error, which was described in the IMLP Registration Phase subsection of the Methods section. As previously stated, this problem forms a GTLS optimization problem that must be solved in the registration phases of the IMLP algorithm and of closely related prior works. We evaluate the proposed GTLS method on the basis of efficiency, accuracy, and stability relative to the prior methods proposed for solving this problem by Estepar et al. [10] and Balachandran and Fitzpatrick [31]. These results are also compared to the closed-form, least-squares solution for the isotropic noise case [26], which constitutes the registration phase of the standard ICP algorithm.

Each method was evaluated using a Matlab-based implementation. For the method of Balachandran and Fitzpatrick, we use the Matlab source code included in their paper [31]. For the other methods we have created our own Matlab implementations, including an implementation of the rotation estimation method of Ohta and Kanatani [28], which is a sub-component of the method by Estepar et al.

A high degree of instability was initially encountered when using the method of Estepar et al. with large translational offsets. We found that a small modification sufficed to fix the issue, which involved applying their translation estimate prior to the first estimate for rotation. This modification was used throughout our study.

As previously noted, one limitation of the method by Balachandran and Fitzpatrick is that this method employs a single noise covariance that remains fixed as the algorithm iterates, due to the assumption of anisotropic noise in only one point set. Although noise in both point sets may be initially considered by combining the noise models to form a single covariance prior to calling their method (as in the covariance expressions of (9) for example), in this case the accuracy of the method still diminishes relative to the magnitude of rotational misalignment because the effective noise covariance is not updated as the method iterates.

Because of this limitation, we conduct a two-part study. The first study (Experiment 1A) investigates anisotropic noise present in both the source and target point sets. The second study (Experiment 1B) investigates anisotropic noise present in only the target point set with isotropic noise present in the source point set. For the second study, the assumption of a fixed effective covariance becomes correct, since a change in the orientation of the source points has no impact. We have included the method of Balachandran and Fitzpatrick in the evaluation of both studies, while computing an effective noise covariance as described in the foregoing paragraph.

The method of Balachandran and Fitzpatrick specifies initializing the anisotropic registration with the isotropic-noise solution before optimizing with respect to the GTLS cost function. We have performed a portion of the experiments both with and without isotropic initialization applied prior to each GTLS method. In order to better investigate the merit of the numerical machinery behind each GTLS approach, isotropic initialization was not used in Experiment 1A. In order to investigate the impact of initialization on each GTLS method, experiments were conducted both with and without isotropic initialization in Experiment 1B.

In order to compare all methods on level ground, several concerns had to be addressed. The first concern regards the termination criteria used by each method, which was defined (or modified) to be when the magnitude of change in the estimated transformation parameters falls below 0.0001 mm and 0.0001 degrees or when the number of iterations exceeds 60. For the method of Estepar et al., a maximum iteration threshold of 20 was applied to the inner loop (i.e. to the rotation estimation component employing the method of Ohta and Kanatani) while the full outer loop was assigned the same maximum iteration threshold as the other GTLS methods. In practice, we found that these maximum iteration thresholds were only reached under the condition of instability; thus, the iteration threshold was also used to automatically detect and count the occurrence of instability for each method.

The next concern regards the form of input afforded to each method. Every GTLS method compared requires some form of decomposition to be performed on the covariance matrices that define the anisotropic noise model, and these decompositions differ between the methods. To provide equal treatment, we use the noise covariances as base-line input for each GTLS method. Since the implementation by Balachandran and Fitzpatrick was programmed to use pre-computed decompositions (i.e. the inverse square root) of the covariance matrices as input, we have added the required calculation to their method and changed the input to use the covariance matrices directly.

Another concern affecting runtime performance regards the style of Matlab coding. To obtain the best possible runtime performance from each method, all matrix operations were fully vectorized in Matlab code, with the only exception being that a loop over the number of points-pairs was required in order to compute the inverse square root of the covariance matrices for the method of Balachandran and Fitzpatrick, as no solution was identified to fully vectorize this operation across all point-pairs. We have normalized for the runtime impact of this loop in Experiment 1B, which compares the method of Balachandran and Fitzpatrick on its own turf (i.e. with anisotropic noise in only one point set), by using a loop to compute the covariance decompositions required by the other GTLS methods as well. This loop-normalization was not performed for Experiment 1A, however, as the runtime comparison with Balachandran and Fitzpatrick is already largely incongruent for that study due to their assumption of a fixed covariance (i.e. anisotropic noise in only one point set), whereas the other GTLS methods re-compute the covariance decompositions in every iteration. Another reason that fully vectorized implementations are used in Experiment 1A is in order to assess the full potential of the other methods.

As a final leveling of the playing field, a runtime normalization was applied in Experiment 1B for the assumption of a fixed covariance (i.e. anisotropic noise in only one point set). This was accomplished by creating variants of the implementations of the proposed Gauss-Newton-based method and of the method by Estepar et al. that assume, like the method of Balachandran and Fitzpatrick, that the effective noise covariance remains fixed for any orientation of the source point set. This test therefore provides a reasonable relative comparison of the runtimes that can be expected from each of the various GTLS optimization schemes.

Experiments for the various studies comprising Experiment 1 were conducted by first generating two noisy point sets with known correspondence and known ground-truth alignment, second applying a random misalignment between the point sets, and third registering the point sets using each registration method. To form a pair of corresponding point sets, a set of 50 points was randomly generated being uniformly distributed within the interval [-100, 100] mm along each dimension in 3D space. These points served as the ground-truth points and also provided the ground-truth alignment of the two point sets. From this single set of ground-truth points, two different noisy point sets were generated by addition of zero-mean, multivariate, Gaussian noise, while using a different covariance for each point set. The two points generated from each ground-truth point were assigned as correspondences between the two point sets. The covariances were generated at random by forming a diagonal matrix of eigenvalues and multiplying on either side by a random rotation and its transpose

$$\begin{array}{c} \text{Random}\;\text{Covariance}=R\;\mathrm{diag}\left(\lambda_{1},\lambda_{2},\lambda_{3}\right)R^{T}\;. \end{array}$$ (29)

In Experiment 1A, involving anisotropic noise in both point sets, the eigenvalues of the noise covariances were set equal to [0.5, 0.5, 2] mm^2^, with different random rotations being used for each set of points. In Experiment 1B, involving anisotropic noise in only one (the target) point set, these same eigenvalues were used for noising the target points, whereas isotropic noise was generated for the source points by setting all eigenvalues equal to 0.25 mm^2^.

For each study, the randomized trials were divided into several bins according to the magnitude of initial misalignment in translation and rotation. For each bin, 1000 randomized trials were performed and the results were averaged. For every trial, different sets of points, noise models, and misalignment were randomly generated and identically applied to each registration method. Registration accuracy was evaluated by computing the average distance between the un-noised point correspondences following each registration. We report this value as the registration error (RE).

The results of Experiment 1A, which incorporates anisotropic noise in both point sets, are presented in Table 1. In this study, two levels of translational misalignment were investigated on the intervals [10, 20] mm and [90, 100] mm, along with five cases of rotational misalignment, which as a group covered the entire interval of [0, 180] degrees.

**Table 1:** Rigid-body registration results for corresponding point sets with anisotropic noise present in both sets of points. (Experiment 1A).

**Trans. (mm)**	**[10, 20]**					**[90, 100]**				
**Rot. (deg.)**	**Alg.**	**Iter.**	**Runtime**	**RE**	**Inst.**	**Alg.**	**Iter.**	**Runtime**	**RE**	**Inst.**
[0, 15]	Isotropic	1.0	0.0001	0.439	0	Isotropic	1.0	0.0001	0.442	0
	Estepar	15.1	0.0106	0.423	0	Estepar	15.1	0.0106	0.424	0
	Balach.	28.1	0.0060	0.423	0	Balach.	32.9	0.0068	0.424	0
	Proposed	3.8	0.0014	0.422	0	Proposed	3.8	0.0013	0.423	0
[15, 45]	Isotropic	1.0	0.0001	0.443	0	Isotropic	1.0	0.0001	0.442	0
	Estepar	17.2	0.0120	0.432	0	Estepar	17.2	0.0120	0.431	0
	Balach.	34.1	0.0070	0.428	0	Balach.	37.5	0.0076	0.427	0
	Proposed	4.4	0.0015	0.424	0	Proposed	4.4	0.0015	0.423	0
[45, 90]	Isotropic	1.0	0.0001	0.442	0	Isotropic	1.0	0.0001	0.435	0
	Estepar	18.8	0.0131	0.456	0	Estepar	18.8	0.0130	0.450	0
	Balach.	38.1	0.0078	0.442	0	Balach.	40.3	0.0081	0.436	0
	Proposed	5.1	0.0017	0.424	0	Proposed	5.1	0.0017	0.416	0
[90, 150]	Isotropic	1.0	0.0001	0.446	0	Isotropic	1.0	0.0001	0.439	0
	Estepar	22.9	0.0154	0.469	2	Estepar	23.3	0.0157	0.466	2
	Balach.	39.4	0.0080	0.448	0	Balach.	41.9	0.0084	0.444	0
	Proposed	6.3	0.0021	0.430	0	Proposed	6.3	0.0021	0.421	0
[150, 180]	Isotropic	1.0	0.0001	0.444	0	Isotropic	1.0	0.0001	0.442	0
	Estepar	28.0	0.0184	0.475	60	Estepar	27.6	0.0181	0.477	59
	Balach.	42.0	0.0085	0.439	0	Balach.	44.5	0.0088	0.441	0
	Proposed	8.8	0.0028	0.424	0	Proposed	8.7	0.0028	0.426	0

Results report the efficiency (number of iterations and runtime (seconds)), registration error (RE) (mm), and instability (% of trials) of the GTLS registration method proposed in this paper compared to the closed-form isotropic solution [26] and the prior GTLS methods of Estepar et al. [10] and Balachandran and Fitzpatrick [31]. The tests are binned according to the magnitude of initial misalignment in translation (mm) and rotation (degrees); each bin represents average values measured over 1000 randomized trials.

As seen in the results, the proposed Gauss-Newton-based method achieves lower registration error than all compared methods across all test cases. The proposed method also maintains consistent registration error across all misalignments studied, achieving significant improvement with respect to the isotropic solution in every case. In contrast, the prior anisotropic methods of Estepar et al. and of Balachandran and Fitzpatrick worsen in accuracy as rotational misalignment increases and tend to provide larger registration errors than even the isotropic solution for rotational misalignments on the interval [45, 90] degrees and beyond.

The proposed method’s runtime is also several times more efficient than the other anisotropic solutions; computing a solution requires much fewer iterations (4–9) compared to the methods of Estepar et al. (15–28) and Balachandran and Fitzpatrick (28–45). Note that the iteration count for the method of Estepar et al. is reported as the total number of evaluations of its inner loop, which is where the vast majority of computation takes place for that method.

Another significant observation regarding the results of Experimant 1A is that the proposed method and that of Balachandran and Fitzpatrick are stable under all conditions tested, whereas the method of Estepar et al. encounters frequent instability for large rotational misalignment, with the portion of unstable trials reaching 60% for the largest rotation interval of [150, 180] degrees.

For the method of Balachandrian and Fitzpatrick, the increase in registration error with respect to rotation is understood to result from the assumption of a constant noise covariance as described earlier. However, it is not clear why the method of Estepar et al. exhibits a similar issue. To shed more light on this and on the issue of instability we include a third study (Experiment 1C) in this section.

Table 2 presents the results of Experiment 1B, which incorporates anisotropic noise in only one point set and isotropic noise in the other. In this study, translational misalignment is limited to a large interval of [90, 100] mm, while the test cases for rotational misalignment remain unchanged. The trials for this experiment are conducted twice: once with and once without initializing the anisotropic methods to the isotropic noise solution; other test conditions (the exact point sets, noise, etc.) remain identical between the two types of trials.

**Table 2:** Rigid-body registration results for corresponding point sets with anisotropic noise present in one set of points and isotropic noise present in the other. (Experiment 1B).

	**Without Isotropic Initialization**					**With Isotropic Initialization**				
**Rot. (deg.)**	**Alg.**	**Iter.**	**Runtime**	**RE**	**Inst.**	**Alg.**	**Iter.**	**Runtime**	**RE**	**Inst.**
[0, 15]	Isotropic	1.0	0.0001	0.349	0	Isotropic	-	-	-	-
	Estepar	14.6	0.0100	0.332	0	Estepar	10.4	0.0080	0.332	0
	Balach.	32.9	0.0068	0.333	0	Balach.	14.8	0.0039	0.332	0
	Proposed	3.7	0.0011	0.332	0	Proposed	2.9	0.0012	0.332	0
[15, 45]	Isotropic	1.0	0.0001	0.347	0	Isotropic	1	0.0001	0.347	0
	Estepar	17.1	0.0115	0.338	0	Estepar	10.8	0.0076	0.338	0
	Balach.	37.5	0.0076	0.330	0	Balach.	14.6	0.0036	0.329	0
	Proposed	4.2	0.0012	0.330	0	Proposed	2.9	0.0011	0.330	0
[45, 90]	Isotropic	1.0	0.0001	0.341	0	Isotropic	1	0.0001	0.341	0
	Estepar	18.7	0.0125	0.352	0	Estepar	10.8	0.0077	0.352	0
	Balach.	40.2	0.0081	0.325	0	Balach.	14.4	0.0037	0.325	0
	Proposed	5.0	0.0013	0.325	0	Proposed	2.9	0.0011	0.325	0
[90, 150]	Isotropic	1.0	0.0001	0.345	0	Isotropic	1	0.0001	0.345	0
	Estepar	22.6	0.0149	0.366	2	Estepar	11.8	0.0082	0.365	0
	Balach.	41.9	0.0084	0.330	0	Balach.	14.6	0.0037	0.330	0
	Proposed	6.1	0.0015	0.330	0	Proposed	2.9	0.0011	0.330	0
[150, 180]	Isotropic	1.0	0.0001	0.350	0	Isotropic	1	0.0001	0.350	0
	Estepar	26.7	0.0173	0.373	60	Estepar	16.2	0.0109	0.385	10
	Balach.	44.5	0.0089	0.333	0	Balach.	14.5	0.0037	0.333	0
	Proposed	8.5	0.0018	0.333	0	Proposed	2.9	0.0011	0.333	0

Results report the efficiency (number of iterations and runtime (seconds)), registration error (RE) (mm), and instability (% of trials) of the GTLS method proposed in this paper compared to the closed-form isotropic solution [26] and the prior GTLS methods of Estepar et al. [10] and Balachandran and Fitzpatrick [31]. The tests are binned according to the magnitude of initial misalignment in rotation (degrees), with all bins having a translational misalignment in the range [90, 100] mm; each bin represents average values measured over 1000 randomized trials.

As seen in Table 2, the outcome is similar to the earlier study, with the most notable difference being that the method of Balachandran and Fitzpatrick computes an equally accurate registration as the proposed method, which confirms that the high registration errors encountered for this method in the prior study resulted from its assumption of anisotropic noise in only one point set. Concerning the method of Estepar et al., the increase in registration error with respect to rotational misalignment remains, which indicates a different source of error for this method.

It is also interesting to note, concerning the method of Estepar et al., that although the occurrence of unstable trials is reduced by initialization to the isotropic solution, the problem of instability does not go away at the largest range of rotational misalignment.

The proposed method remains largely more efficient than the other anisotropic methods, both with and without initialization to the isotropic solution. It is interesting to note that initialization to the isotropic solution approximately halves the runtime of the method by Balachandran and Fitzpatrick whereas the runtime for the proposed method is reduced by much less, even though the relative decrease in the number of iterations for each method is more similar. This observation indicates that the proposed method has low computational complexity beyond that of computing the initial covariance decompositions (recall for this study that the effective covariances are assumed to be constant), whereas the method of Balachandran and Fitzpatrick retains significantly more overhead per iteration following the initial covariance decompositions. Isotropic initialization also significantly reduces the runtime of the method by Estepar et al. to a little more than half its value otherwise.

Table 3 presents the results of the final study in this series, Experiment 1C, which is intended to further investigate the registration error and instability issues encountered by the method of Estepar et al. relative to increases in rotational offset. This study evaluates only the rotational component of their method. That is, comparison is made concerning the GTLS rotational estimation method of Ohta and Kanatani [28] relative to the proposed Gauss-Newton-based GTLS method and relative to the rotation computed under an isotropic noise assumption. For this comparison, the proposed method and the isotropic solution were modified to estimate only parameters of rotation, assuming translation to be zero. This study was conducted in similar fashion as Experiment 1A, except that only rotational misalignment was applied between the two point sets.

**Table 3:** Rotation-only registration results for corresponding point sets with anisotropic noise present in both sets of points. (Experiment 1C).

**Rot. (deg.)**	**Alg.**	**Iter.**	**Runtime**	**RE**	**Inst.**
[0, 15]	Isotropic	1.0	0.0000	0.304	0
	Kanatani	4.0	0.0025	0.279	0
	Proposed	3.8	0.0013	0.278	0
[15, 45]	Isotropic	1.0	0.0000	0.292	0
	Kanatani	4.0	0.0025	0.283	0
	Proposed	4.4	0.0015	0.269	0
[45, 90]	Isotropic	1.0	0.0000	0.295	0
	Kanatani	4.0	0.0025	0.313	0
	Proposed	5.1	0.0017	0.271	0
[90, 150]	Isotropic	1.0	0.0000	0.292	0
	Kanatani	4.5	0.0028	0.323	0
	Proposed	6.3	0.0020	0.265	0
[150, 180]	Isotropic	1.0	0.0000	0.286	0
	Kanatani	6.1	0.0038	0.360	10
	Proposed	8.7	0.0028	0.263	0

Results report the efficiency (number of iterations and runtime (seconds)), registration error (RE) (mm), and instability (% of trials) of the GTLS method proposed in this paper (modified to computer only rotation) compared to the closed-form isotropic solution [26] and the prior GTLS rotation estimation method of Ohta and Kanatani [28]. The tests are binned according to the magnitude of initial misalignment in rotation (degrees) with translational misalignment being zero; each bin represents average values measured over 1000 randomized trials.

As seen in Table 3, the method of Ohta and Kanatani exhibits the same increase in registration error relative to rotational misalignment as encountered by the method of Estepar et al. Further, the rotation estimation exhibits similar instability under large rotational misalignment. This indicates that a source of error and instability for the method of Estepar et al. lies in the rotation estimation component.

The rotation estimation method in this case uses a quaternion parameterization for rotation that is optimized by applying the renormalization method of Kanatani [29]. Matei and Meer [30] have presented a technique called heteroscedastic errors-in-variables (HEIV) estimator, which is closely related to the renormalization method of Kanatani, in which they present a similarly parameterized method for computing the full rigid-body alignment of two point sets under anisotropic noise. In their work, they point out a discontinuity in the quaternion representation for rotation that produces instability for rotational misalignments close to +/- 180 degrees. It is possible that the rotation estimation method of Kanatani suffers from a similar issue, though we have not verified this further.

### Experiment 2: Registering a Mesh Target

**Fig 1:**
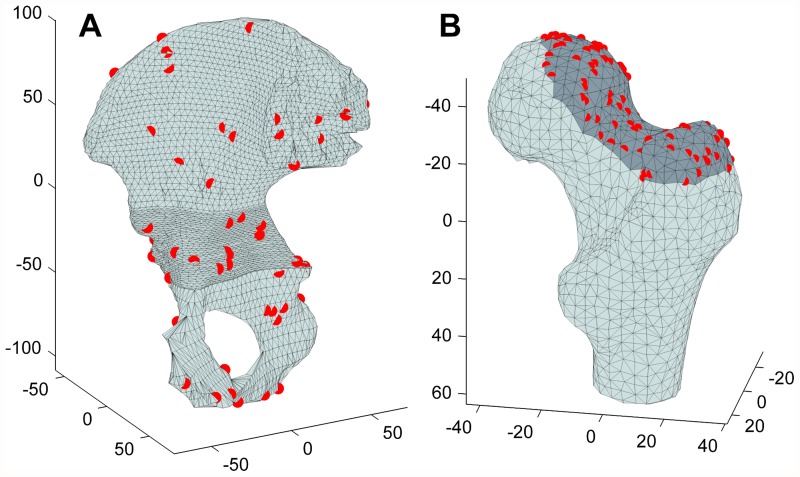
Human hip- and femur-bone meshes used in the registration studies. The red points represent a typical randomly generated source shape as sampled from the mesh surface. (A): The hip mesh is used in registration Experiments 2–5. (B): The femur mesh is used for the sub-shape registration study of Experiment 6, where points for the source shape are sampled from the shaded region of the mesh.

In this study, we evaluate the performance of the IMLP algorithm for registering a target shape represented by a triangular mesh. The experiment is divided into two sub-experiments (Experiments 2A and 2B) in order to evaluate the algorithm’s performance under different magnitudes of shape misalignment. The shape being registered in both cases is a human hip model segmented from CT imaging to form a surface mesh (Fig. 1A).

The experiments are conducted by randomly generating a set of 100 noisy points from the mesh surface to form a source shape and applying a random misalignment between the source shape and the mesh. The source points are then registered back to the mesh, which forms the target shape. This approach enables accurate assessment of the registration error under varying noise conditions, since both the ground-truth alignment and the generative noise models are known.

Nine different test cases were conducted to evaluate each of the nine different noise models defined in Table 4, which specifies the variance of multivariate Gaussian noise generated in the surface-normal vs. surface-parallel directions at each source point. Thus, the noise applied to each source point was conditioned relative to the orientation of the surface at that point. The first three test cases apply isotropic noise, the next three tests cases apply anisotropic noise of high surface-normal variance, and the final three test cases apply anisotropic noise of high surface-parallel variance, each in order of increasing magnitude of variance and increasing anisotropy. Within each of the nine test cases, 300 randomized registration trials were conducted, each involving new randomly generated points, noise, and misalignment. All compared algorithms were executed once per trial under identical test conditions (i.e. identical shape, noise, and misalignment) as generated for that trial.

**Table 4:** Generative noise models (test cases) used in the randomized registration trials of Experiments 2–5.

**Test Case**	**1**	**2**	**3**	**4**	**5**	**6**	**7**	**8**	**9**
**Surface-Normal Std. Dev. (mm)**	0.5	1.0	2.0	1.0	2.0	2.0	0.5	1.0	0.5
**Surface-Parallel Std. Dev. (mm)**	0.5	1.0	2.0	0.5	1.0	0.5	1.0	2.0	2.0

This table defines the standard deviation of noise generated in the surface-normal and surface-parallel directions for each point of the source shapes in Experiments 2–5.

Registration errors were measured by randomly sampling a set of 100 non-noisy points from the mesh surface to be used in validation. Following registration, the average distance between the registered and known ground-truth positions of the validation points is measured and recorded as the target registration error (TRE). The average TRE is then reported within each test case for each algorithm. To prevent TRE outliers from skewing the averages, only successful registrations are included in the reported TRE averages. A registration trial is considered successful if the TRE is within 10 mm. The number of registration failures for each algorithm is also counted and reported within each test case. This procedure is followed for all the registration studies that follow in this paper.

The entire experiment was conducted twice for two different intervals of random initial misalignment: once for the misalignment interval of [15, 30] mm translation and [15, 30] degrees rotation (Experiment 2A) and again for the misalignment interval of [30, 60] mm translation and [30, 60] degrees rotation (Experiment 2B). These random misalignments were generated along random translational directions and random rotational axes.

This experiment compares the algorithms of standard ICP [1], IMLP, and the two variants IMLP-CP and IMLP-MD, which, as described in the introduction to the Results and Discussion section, provide near comparison to the GTLS-ICP [10] and A-ICP [12] algorithms, respectively. For IMLP (and variants), the measurement-noise covariances of the source points were set to correspond with the generative noise models defined in Table 4, while the measurement covariances for the target shape were set to zero, since no noise was added to the mesh. Because the mesh fully represents the continuous surface of the target, IMLP’s (and variants’) surface-model covariances were set to zero, as these are intended for registering non-continuous (i.e. point-cloud) surface representations. Outlier detection was disabled by setting IMLP’s (and variants’) chi-square inverse CDF threshold ($\chi_{\text{thresh}}^{2}$) to a large value.

**Fig 2:**
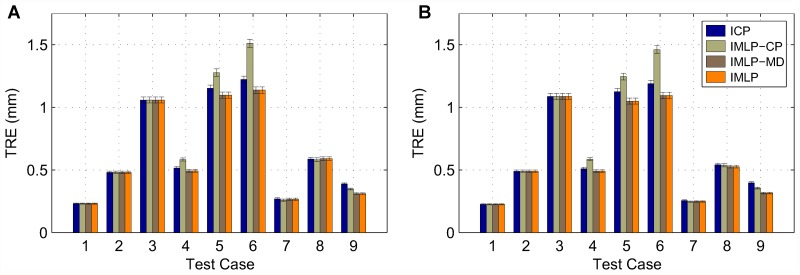
Registration errors for registering a mesh target shape. (Experiment 2). Source shapes were randomly generated from a mesh model of a human hip (Fig. 1A), misaligned by (A): [15, 30] mm / degrees and (B): [30, 60] mm / degrees, and registered back to the mesh. The test cases represent the different noise models used to generate noise on the source shape (Table 4). For each test case, 300 randomized trials were conducted, with successful registrations being used to compute an average target registration error (TRE). The error bars provide approximate standard deviations of the reported average TRE values. The proposed IMLP algorithm was evaluated relative to standard ICP [1] and relative to near-comparisons of GTLS-ICP [10] and A-ICP [12] using IMLP-CP and IMLP-MD, which modify IMLP’s most-likely match criteria to that of closest-point and Mahalanobis-distance matching, respectively.

Results for Experiments 2A and 2B are presented in Figs. 2A and 2B for each range of misalignment. Similar results are obtained in both cases. As expected, the average TREs for the first three test cases are identical among all algorithms since, in this case, the noise model of IMLP reduces to the Euclidean-distance computations of standard ICP. For the anisotropic-noise test cases, the IMLP algorithm consistently achieves slightly improved or approximately equivalent registration accuracy compared to ICP. For this study, the Mahalanobis distance matching variant (IMLP-MD) computes approximately the same registration errors as IMLP, whereas the closest-point matching variant (IMLP-CP) generally performs worse, even performing worse than standard ICP for the test cases involving high variance of noise in the surface-normal direction.

The error bars displayed in Fig. 2 (and in other figures throughout this section) are approximations for the standard deviations of each displayed TRE average. They are calculated by computing the standard deviation of the TREs for a given average and dividing by the square root of the number of trials used to compute that average. This is the procedure for calculating the standard deviation of a sample mean for independent identically distributed (iid) Gaussian data [33]. We have verified that the histograms of TREs for successful trials reasonably resemble those of Gaussian distributions, in general; thus, the displayed standard deviations may be considered as close approximations.

Table 5 lists the percentage of registration failures for each algorithm and test case in Experiment 2B. For Experiment 2A, the failure rates were 0% in all cases. As seen in the table, the algorithms performed near equally with a maximum overall failure rate of 2%.

**Table 5:** Registration failure rates for registering a mesh target shape. (Experiment 2B).

**Alg.**	**Failure Rate (%) by Test Case**								
	**1**	**2**	**3**	**4**	**5**	**6**	**7**	**8**	**9**
ICP	0.3	0.7	0.3	1.0	0.3	2.0	1.0	0.7	0.3
IMLP-CP	0.3	0.7	0.3	1.0	0.3	2.0	1.0	0.7	0.7
IMLP-MD	0.3	0.7	0.3	1.0	0.3	2.0	1.0	0.7	0.7
IMLP	0.3	0.7	0.3	1.0	0.3	2.0	1.0	0.7	0.7

Source shapes were randomly generated from a mesh model of a human hip (Fig. 1A), misaligned by [15, 30] mm / degrees in (Experiment 2A) and [30, 60] mm / degrees in (Experiment 2B), and registered directly back to the mesh. The test cases represent the different noise models used to generate noise on the source shape (Table 4). For each test case, 300 randomized trials were conducted, with the percent of unsuccessful registrations (TRE > 10 mm) being shown in this table. The proposed IMLP algorithm was evaluated relative to standard ICP [1] and relative to near-comparisons of GTLS-ICP [10] and A-ICP [12] using IMLP-CP and IMLP-MD, which modify IMLPâs most-likely match criteria to that of closest-point and Mahalanobis-distance matching, respectively. Failure rates for Experiment 2A (which are not shown in this table) were 0% for all algorithms and test cases.

Table 6 presents a runtime analysis for the successful registrations within each experiment. Due to its greater complexity, IMLP has a runtime of approximately 3.5x that of ICP on average.

**Table 6:** Runtimes for registering a mesh target shape. (Experiment 2).

**Exp.**	**Alg.**	**Average Runtimes (sec.) by Test Case**								
		**1**	**2**	**3**	**4**	**5**	**6**	**7**	**8**	**9**
2A	ICP	0.086	0.094	0.109	0.107	0.119	0.115	0.087	0.097	0.095
	IMLP-CP	0.194	0.204	0.228	0.252	0.287	0.263	0.138	0.157	0.118
	IMLP-MD	0.270	0.299	0.323	0.389	0.437	0.401	0.226	0.249	0.219
	IMLP	0.352	0.377	0.407	0.499	0.543	0.569	0.258	0.239	0.202
2B	ICP	0.142	0.147	0.165	0.145	0.16	0.168	0.135	0.142	0.166
	IMLP-CP	0.285	0.286	0.322	0.346	0.351	0.343	0.221	0.222	0.196
	IMLP-MD	0.339	0.372	0.381	0.462	0.496	0.467	0.298	0.311	0.275
	IMLP	0.444	0.454	0.495	0.588	0.638	0.686	0.425	0.480	0.379

Average runtimes of successful registrations from Experiment 2 are reported, where 300 randomized trials were conducted for each test case. Each test case represents a different generative noise model (Table 4) applied to points of the source shape. Results are reported for initial shape misalignments of [15, 30] mm / degrees (Experiment 2A) and [30, 60] mm / degrees (Experiment 2B). The algorithms compared include single-threaded implementations of ICP [1], IMLP, and the two IMLP variants IMLP-CP and IMLP-MD.

### Experiment 3: Registering a Mesh Target with Outliers

Experiment 3 follows the same procedure as Experiment 2, using the same target shape and equivalent test cases (noise models) and numbers of trials. However, in this study the source shape is corrupted with additional points added as outliers. This experiment is again divided into two studies (Experiments 3A and 3B), one for each range of misalignment. Each study is further divided into sub-studies with varying percentages of outliers including 5%, 10%, 20%, and 30% outliers, which we refer to as sub-experiments i-iv, respectively. Since the purpose of this study is to evaluate the merit of IMLP’s outlier mechanism, the IMLP variants (which have the same outlier mechanism) are not evaluated. Robust ICP [4] is also added to the set of compared algorithms.

The additional outlier samples were generated for each trial by randomly selecting points on the mesh surface and projecting each point outward from the mesh by a random distance on the interval [10, 20] mm.

IMLP’s outlier detection was enabled by setting its chi-square inverse CDF threshold ($\chi_{\text{thresh}}^{2}$) to the values of {7.81, 6.25, 4.64, 3.66} for sub-experiments i-iv, respectively. These values correspond to chi-square inverse CDF probabilities of {0.95, 0.9, 0.8, 0.7}, which were chosen to directly correspond to the percentage of outliers in each test case.

For Robust ICP we follow the suggestion of its author and relate the user parameter *D*, which is used to determine when a registration is good, to the resolution of the shape data. This was accomplished by computing the average distance between a triangle center point and that of its neighbors in the mesh (in this case approximately 1.8 mm). We also set the user parameter $D_{\text{max}}^{0}$, which controls the maximum tolerable match distance of the first iteration, to a large value in order to take all matches into consideration in the first iteration, which afforded the algorithm the best chance at computing a successful registration.

**Fig 3:**
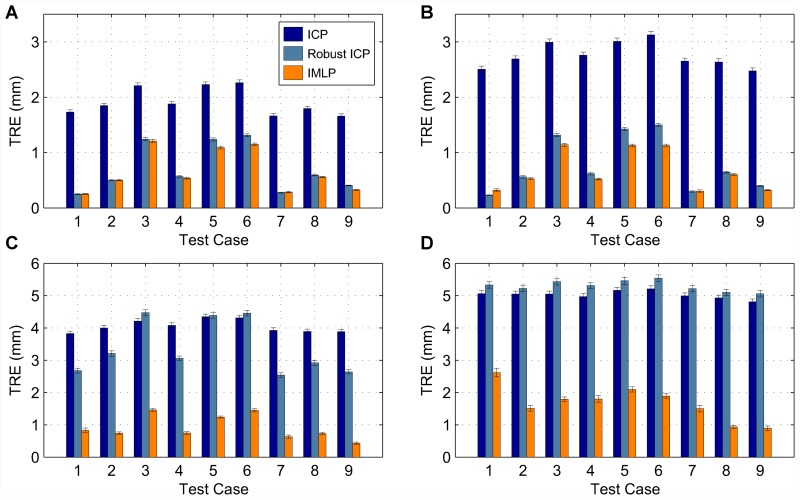
Registration errors for registering a source shape containing outliers to a mesh target under moderate misalignment. (Experiment 3A). Source shapes were randomly generated from the hip mesh (Fig. 1A), misaligned by [15, 30] mm / degrees, and registered back to the mesh. The test cases represent the different noise models used to generate noise on the source shape (Table 4). Outliers were added to the source shape constituting (A): 5%, (B): 10%, (C): 20%, and (D): 30% of the source points. For each test case, 300 randomized trials were conducted, with successful registrations being used to compute an average target registration error (TRE). The error bars provide approximate standard deviations of the reported average TRE values. The proposed IMLP algorithm was evaluated relative to standard ICP [1] and relative to a robust variant of ICP [4].

**Fig 4:**
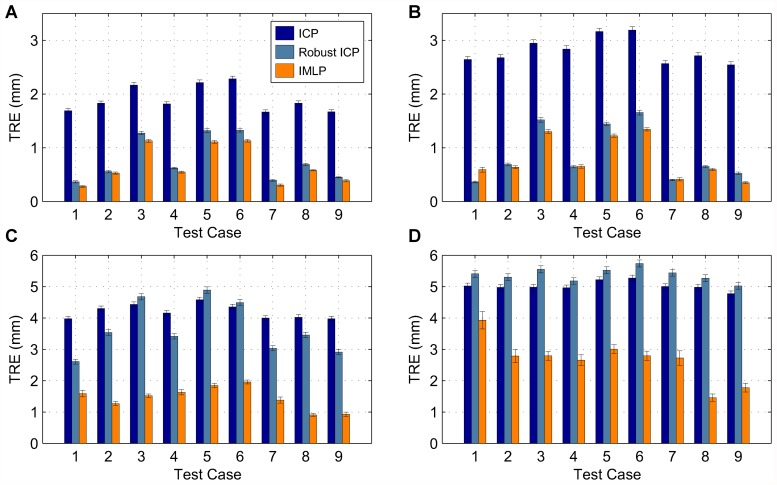
Registration errors for registering a source shape containing outliers to a mesh target under large misalignment. (Experiment 3B). Source shapes were randomly generated from the hip mesh (Fig. 1A), misaligned by [30, 60] mm / degrees, and registered back to the mesh. The test cases represent the different noise models used to generate noise on the source shape (Table 4). Outliers were added to the source shape constituting (A): 5%, (B): 10%, (C): 20%, and (D): 30% of the source points. For each test case, 300 randomized trials were conducted, with successful registrations being used to compute an average target registration error (TRE). The error bars provide approximate standard deviations of the reported average TRE values. The proposed IMLP algorithm was evaluated relative to standard ICP [1] and relative to a robust variant of ICP [4].

The registration accuracy for this study is presented in Figs. 3 and 4 for Experiments 3A and 3B, respectively. Figs. 3 and 4 are each divided into four sub-figures (A-D) corresponding to sub-experiments (i-iv) of Experiments 3A and 3B, respectively, for each level of outliers. The analysis to produce these results was conducted in the same manner as described for Experiment 2. As seen in the figures, IMLP widely outperforms ICP in terms of TRE for all test cases and performs marginally better overall than Robust ICP for 5% and 10% outliers and much better than Robust ICP at higher levels of outliers, where the TRE for Robust ICP approaches and even surpasses that of standard ICP. In contrast, IMLP’s TRE remains fairly stable up to 20% outliers and begins to increase at the 30% level.

The registration failure rates shown in Table 7 for this study indicate that although standard ICP is the worst algorithm in terms of TRE, it has the highest registration success rate. At the lesser misalignment range, the failure rates of all algorithms are below 1% for up to 10% outliers, with IMLP having marginally higher failure rates at the 20% outlier level. At 30% outliers, the failure rate of IMLP increases significantly, accompanied by a marginal increase in the failure rate of Robust ICP. For the large misalignment range a different pattern emerges with Robust ICP exhibiting high failure rates across all outlier levels, whereas IMLP maintains low failure rates for up to 10% outliers. The failure rate of standard ICP increases marginally at this offset range yet still remains quite low.

**Table 7:** Registration failure rates for registering a mesh target shape with outliers. (Experiment 3).

**Exp.**	**Outliers**	**Alg.**	**Failure Rates (%) by Test Case**								
			**1**	**2**	**3**	**4**	**5**	**6**	**7**	**8**	**9**
3A-i	5%	ICP	0.0	0.0	0.0	0.0	0.0	0.0	0.0	0.0	0.0
		Robust ICP	0.0	0.3	0.3	0.0	0.0	0.0	0.3	0.0	0.7
		IMLP	0.0	0.0	0.0	0.0	0.0	0.0	0.3	0.0	0.0
3A-ii	10%	ICP	0.0	0.0	0.0	0.0	0.3	0.0	0.0	0.0	0.0
		Robust ICP	0.0	0.7	0.0	0.0	0.3	0.3	0.0	0.0	0.3
		IMLP	0.0	0.7	0.0	0.3	0.7	0.3	0.3	0.0	0.0
3A-iii	20%	ICP	0.3	0.3	0.7	0.0	0.0	0.3	0.3	0.3	1.0
		Robust ICP	1.0	0.7	1.0	0.7	0.0	1.3	1.3	0.7	1.0
		IMLP	4.0	2.7	4.0	4.3	2.0	1.7	3.7	2.0	3.7
3A-iv	30%	ICP	0.3	0.3	1.7	1.3	1.3	1.0	0.7	0.7	1.0
		Robust ICP	1.0	2.0	3.0	2.7	5.0	3.7	2.7	2.0	1.7
		IMLP	28.0	15.0	11.0	25.0	13.3	9.3	22.3	13.0	13.3
3B-i	5%	ICP	0.3	0.7	0.3	0.3	0.3	1.0	0.0	2.7	0.3
		Robust ICP	12.3	12.3	6.3	5.0	14.0	10.0	9.7	10.0	9.7
		IMLP	1.3	2.0	1.3	1.0	0.7	0.3	0.3	2.3	1.7
3B-ii	10%	ICP	0.7	1.3	0.3	0.3	0.7	1.0	1.0	1.3	1.0
		Robust ICP	11.7	7.7	9.3	11.7	10.0	12.7	8.7	7.0	8.3
		IMLP	3.0	2.0	3.0	5.7	1.3	3.3	2.3	2.3	2.3
3B-iii	20%	ICP	1.7	1.0	0.0	0.7	0.7	1.7	1.0	2.0	0.3
		Robust ICP	9.0	11.3	6.7	8.0	13.0	11.0	9.0	13.0	10.3
		IMLP	26.0	17.0	9.7	18.3	16.3	14.3	26.7	16.3	16.0
3B-iv	30%	ICP	2.7	2.0	2.3	2.0	2.7	1.7	1.3	1.7	2.3
		Robust ICP	12.7	17.3	12.7	12.3	13.7	14.3	10.3	11.3	12.7
		IMLP	75.0	65.3	47.0	62.7	52.3	53.0	75.3	55.3	57.7

Source shapes were randomly generated from a mesh model of a human hip (Fig. 1A), misaligned by [15, 30] mm / degrees in (Experiment 3A) and [30, 60] mm / degrees in (Experiment 3B), and registered back to the mesh. The test cases represent the different noise models used to generate noise on the source shape (Table 4). Outliers were added to the source shape constituting 5% (-i), 10% (-ii), 20% (-iii), and 30% (-iv) of the source points. For each test case, 300 randomized trials were conducted, with the percent of unsuccessful registrations (TRE > 10 mm) being shown in this table. The proposed IMLP algorithm was evaluated relative to standard ICP [1] and relative to a robust variant of ICP [4].

### Experiment 4: Registering a Point-Cloud Target

In this study, we investigate the performance of the IMLP algorithm for registering a target shape represented by a point cloud. Because the registration only involves point-cloud shapes, several additional algorithms can be compared. In this experiment, we evaluate standard ICP [1], GICP [11], CPD [20], IMLP, and the two variants IMLP-CP and IMLP-MD.

A dense point cloud formed from the center points of every triangle in the human hip mesh (Fig. 1A) is used as the target shape. Besides this change, the test conditions remain as described for Experiment 2. This study is likewise divided into two parts representing different random misalignment intervals: [15, 30] mm / degrees (Experiment 4A) and [30, 60] mm / degrees (Experiment 4B).

In contrast to Experiment 2, the surface-model covariances of IMLP (and variants) are enabled in this study and defined to have standard deviations of 0.5 mm in the surface-normal direction and 5 mm in the surface-parallel directions. Recall that these covariances provide a linear local approximation of the unmeasured surface surrounding each sampled point in order to improve registration accuracy. The measurement-error covariances remain as defined in Experiment 2.

The GICP algorithm also employs a local surface model surrounding each sampled point and uses its covariance matrices for this sole purpose. The covariance scaling parameter (*ϵ*) of GICP is set to 0.01, which is equal to the ratio of surface-normal vs. surface-parallel variances defined for IMLP. The surface-model used to evaluate GICP is therefore equivalent to the surface-model used by IMLP, because the optimizations performed by GICP do not change with respect to a global scaling of its covariances. We also tested GICP’s default *ε* value of 0.001, but found 0.01 to provide higher accuracy in this study.

Outlier detection is disabled by setting the chi-square inverse CDF threshold of IMLP to a large value and setting the outlier weight of CPD to zero. The maximum match search distance of GICP is also set to a large value in order to not exclude any matches from consideration. Rigid-body transformation without scaling was selected as the CPD registration method, with the target point cloud being used as the GMM centroids and the source point cloud being used as the data points. This choice of roles was found to be important, as reversing the roles of the source and target points produced substantially higher registration errors.

**Fig 5:**
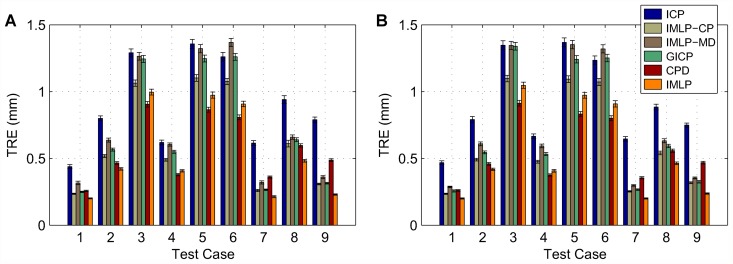
Registration errors for registering a point-cloud target shape. (Experiment 4). Source shapes were randomly generated from a mesh model of a human hip (Fig. 1A), misaligned by (A): [15, 30] mm / degrees and (B): [30, 60] mm / degrees, and registered back to a point-cloud representation of the mesh. The test cases represent different noise models used to generate noise on the source shape (Table 4). For each test case, 300 randomized trials were conducted, with successful registrations being used to compute an average target registration error (TRE). The error bars provide approximate standard deviations of the reported average TRE values. The proposed IMLP algorithm was evaluated relative to standard ICP [1], GICP [11], and CPD [20], as well as relative to near-comparisons of GTLS-ICP [10] and A-ICP [12] using the two variants IMLP-CP and IMLP-MD, which modify IMLP’s most-likely match criteria to that of closest-point and Mahalanobis-distance matching, respectively.

The registration accuracies achieved by each algorithm for this experiment are presented in Fig. 5. Similar results were obtained for both ranges of initial misalignment. As seen in the figure, IMLP achieves significantly better registration accuracy than any other algorithm across all test cases for both ranges of misalignment, with exception of CPD for which IMLP achieves comparatively better accuracy in more than half of the test cases considered. Note that unlike Experiment 2A, in this experiment IMLP strongly outperforms ICP even for the initial test cases involving isotropic measurement noise. The reason for this stems from the surface-model covariances used to model unmeasured surface regions surrounding each sample point.

The advantage of IMLP’s most-likely-point matching criteria is particularly highlighted in comparison to the two variants of IMLP that evaluate modifications of its match criteria, i.e. closest-point matching (IMLP-CP) and Mahalanobis-distance matching (IMLP-MD). IMLP achieves significantly, and in some cases substantially, higher accuracy than either of these variants for all test cases considered. Compared to GICP, IMLP also attains a notable accuracy improvement in all test cases, which further underscores the advantage of IMLP’s most-likely-point matching criteria and of its modeling of measurement error.

It is remarkable that the Mahalanobis-distance matching criteria (IMLP-MD) has worse accuracy in this experiment than closest-point matching (IMLP-CP) and, for some test cases, shows no improvement over standard ICP. This result is surprising, especially given that the reverse was true in Experiment 2A, which involved registering to a mesh rather than a point-cloud target.

Table 8 lists the registration failure rates of each algorithm for the large misalignment range of Experiment 4B. For Experiment 4A, no registration failures were indicated except for standard ICP, which had one failure in the second test case. As shown in the table, all algorithms achieve very low failure rates, with GICP being marginally higher than the others and CPD having the best performance with no registration failures.

**Table 8:** Registration failure rates for registering a point-cloud target shape. (Experiment 4B).

**Alg.**	**Failure Rate (%) by Test Case**								
	**1**	**2**	**3**	**4**	**5**	**6**	**7**	**8**	**9**
ICP	0.3	0.7	0.3	0.7	0.3	2.0	0.7	0.3	0.3
IMLP-CP	0.3	0.7	0.3	0.7	0.3	2.0	0.7	0.3	0.3
IMLP-MD	0.3	0.7	0.3	0.7	0.3	2.0	1.0	0.3	0.3
GICP	1.3	1.3	0.7	2.7	3.7	2.0	2.0	1.3	1.7
CPD	0.0	0.0	0.0	0.0	0.0	0.0	0.0	0.0	0.0
IMLP	0.3	0.7	0.3	0.7	0.3	2.0	1.0	0.3	0.3

Source shapes were randomly generated from a mesh model of a human hip (Fig. 1A), misaligned by [15, 30] mm / degrees in (Experiment 4A) and [30, 60] mm / degrees in (Experiment 4B), and registered back to a point-cloud representation of the mesh. The test cases represent different noise models used to generate noise on the source shape (Table 4). For each test case, 300 randomized trials were conducted, with the percent of unsuccessful registrations (TRE > 10 mm) being shown in this table. The proposed IMLP algorithm was evaluated relative to standard ICP [1], GICP [11], and CPD [20], as well as relative to near-comparisons of GTLS-ICP [10] and A-ICP [12] using IMLP-CP and IMLP-MD, which modify IMLP’s most-likely match criteria to that of closest-point and Mahalanobis-distance matching, respectively. Failure rates for Experiment 4A (which are not shown in the table) were 0% for all algorithms and test cases, except for test case 2, where standard ICP incurred one registration failure.

Table 9 presents a runtime comparison of each algorithm. Standard ICP is the most efficient algorithm, with IMLP-CP coming second at approximately twice the runtime on average. While not shown in the table, the runtime of GICP is also on-par with that of IMLP-CP. The runtime of GICP is excluded from the table because it was executed within a Live Linux distribution running on a USB flash drive with persistent storage, which occasionally stuttered during execution causing inflated runtime averages. Although IMLP’s runtime is approximately 9 times that of standard ICP in this study, IMLP is up to 60 times more efficient than CPD and 45 times more efficient on average. Using the input settings applied to CPD in this study, it was observed that CPD utilized 100% of both cores on the dual-core test platform, unlike the other algorithms which ran single-threaded. Thus, after normalizing for multithreading, IMLP is approximately two orders of magnitude more efficient than CPD.

**Table 9:** Runtimes for registering a point-cloud target shape. (Experiment 4).

**Exp.**	**Alg.**	**Average Runtimes (sec.) by Test Case**								
		**1**	**2**	**3**	**4**	**5**	**6**	**7**	**8**	**9**
4A	ICP	0.009	0.009	0.010	0.009	0.010	0.009	0.009	0.009	0.009
	IMLP-CP	0.015	0.016	0.019	0.016	0.020	0.019	0.015	0.017	0.015
	IMLP-MD	0.068	0.078	0.093	0.079	0.097	0.093	0.067	0.079	0.069
	GICP	-	-	-	-	-	-	-	-	-
	CPD (2 cores)	3.465	4.346	4.336	3.864	4.340	4.374	4.238	4.650	4.484
	IMLP	0.068	0.082	0.102	0.078	0.103	0.099	0.067	0.084	0.073
4B	ICP	0.013	0.013	0.013	0.013	0.013	0.013	0.012	0.012	0.013
	IMLP-CP	0.023	0.025	0.028	0.025	0.028	0.028	0.024	0.025	0.024
	IMLP-MD	0.100	0.109	0.126	0.112	0.127	0.129	0.100	0.109	0.099
	GICP	-	-	-	-	-	-	-	-	-
	CPD (2 cores)	3.584	4.408	4.490	4.279	4.327	4.545	4.378	4.731	4.874
	IMLP	0.101	0.111	0.134	0.115	0.136	0.133	0.103	0.118	0.106

Average runtimes of successful registrations from Experiment 4 are reported, where 300 randomized trials were conducted for each test case. Each test case represents a different generative noise model (Table 4) applied to points of the source shape. Results are also reported for initial shape misalignments of [15, 30] mm / degrees (Experiment 4A) and [30, 60] mm / degrees (Experiment 4B). The algorithms compared include single-threaded implementations of ICP [1], GICP [11], IMLP, and the two IMLP variants IMLP-CP and IMLP-MD. A multi-threaded implementation of CPD [20] is also reported, which made full utilization of 2 cores.

In this study, the runtime difference between ICP and IMLP is greater than observed in Experiment 2A regarding a mesh target. This happens because the node search of the correspondence phase is simplified in this study by not having to compute the closest point on a triangle when computing the distance to a single datum in the PD tree. Although this provides a performance boost to both algorithms, the effect on ICP is much more pronounced since this computation occupies a greater percentage of ICP’s overall runtime.

### Experiment 5: Registering a Point-Cloud Target with Outliers

Experiment 5 follows a similar test scenario as Experiment 4, except that the source point set is corrupted with additional points added as outliers. These outliers are generated in the same manner as Experiment 3. Likewise, this experiment is divided into two studies (Experiments 5A and 5B) corresponding to each range of misalignment and further sub-divided for different percentages of outliers including 5%, 10%, 20%, and 30%, which are referred to as sub-experiments i-iv, respectively. As in Experiment 3, the two variants on IMLP are not included in this outlier study, whereas Robust ICP is added.

For IMLP the chi-square inverse CDF threshold is set according to the percentage of outliers as previously described in Experiment 3. Both the surface-model and measurement-error covariances are used in this study in the same manner as was described in Experiment 4. The user-defined parameters for Robust ICP are also configured as in Experiment 3. Following the lead of CPDâs authors, we set the outlier weight to 0.5. In this case, the target point cloud is assigned as the data points and the source point cloud as the GMM centroids, which is the reverse of Experiment 3, as it was observed that this setting produced substantially lower registration error for the case of non-zero outlier weighting. Concerning the GICP algorithm, although a user-defined parameter is provided for limiting the match-search distance, this mechanism is intended for partial-shape registration rather than outlier handling. Although limiting the match-search distance to 10 mm (to eliminate the outliers positioned between 10 and 20 mm from the surface) improves the registration accuracy for some trials, this also causes registration failure in most cases. Thus, GICP is compared in this study by setting its maximum search distance to a large value and considering it to be a non-robust algorithm.

**Fig 6:**
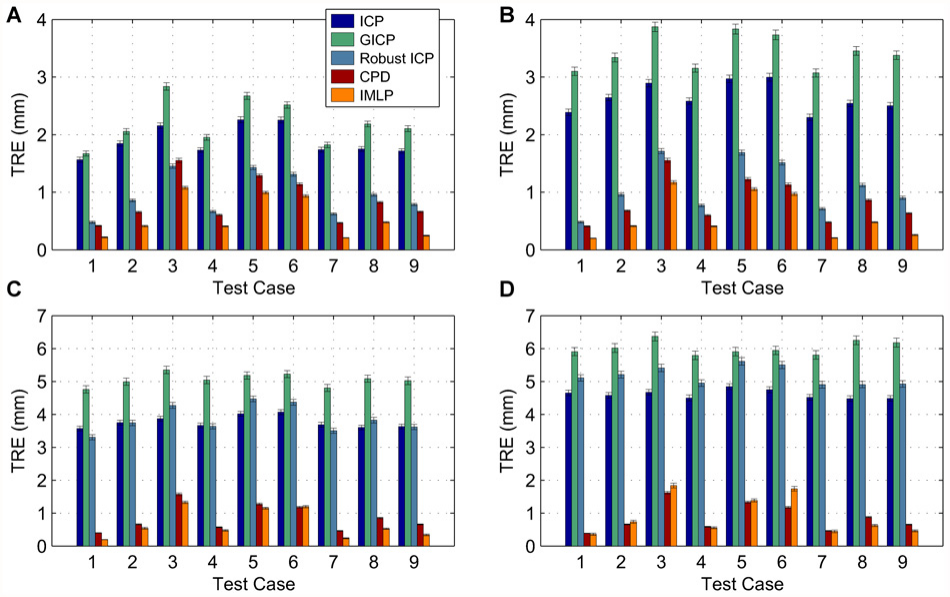
Registration errors for registering a source shape containing outliers to a point-cloud target under moderate misalignment. (Experiment 5A). Source shapes were randomly generated from a mesh model of a human hip (Fig. 1A), misaligned by [15, 30] mm / degrees, and registered back to a point-cloud representation of the mesh. The test cases represent different noise models used to generate noise on the source shape (Table 4). Outliers were added to the source shape constituting (A): 5%, (B): 10%, (C): 20%, and (D): 30% of the source points. For each test case, 300 randomized trials were conducted, with successful registrations being used to compute an average target registration error (TRE). The error bars provide approximate standard deviations of the reported average TRE values. The proposed IMLP algorithm was evaluated relative to standard ICP [1], GICP [11], a robust variant of ICP [4], and CPD [20].

**Fig 7:**
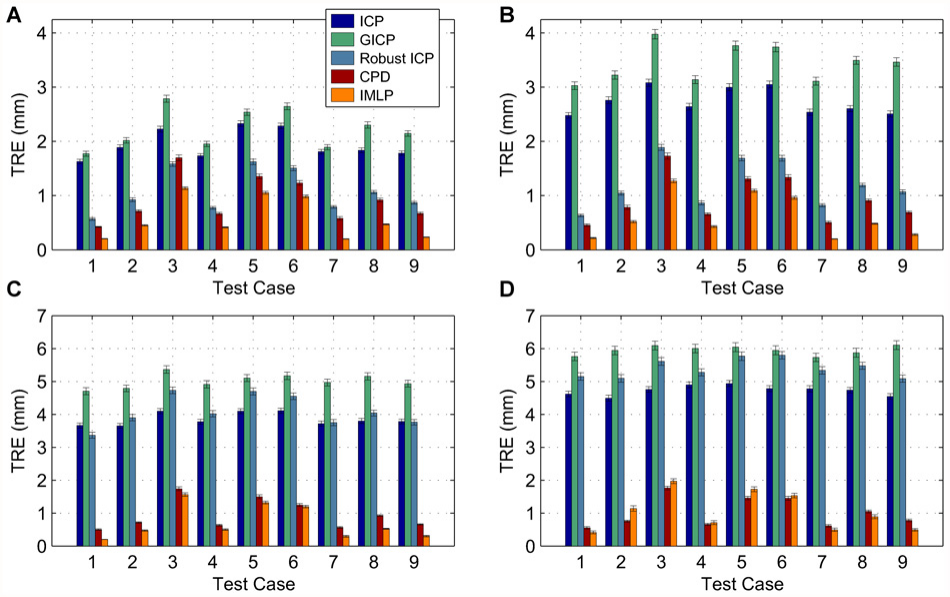
Registration errors for registering a source shape containing outliers to a point-cloud target under large misalignment. (Experiment 5B). Source shapes were randomly generated from a mesh model of a human hip (Fig. 1A), misaligned by [30, 60] mm / degrees, and registered back to a point-cloud representation of the mesh. The test cases represent different noise models used to generate noise on the source shape (Table 4). Outliers were added to the source shape constituting (A): 5%, (B): 10%, (C): 20%, and (D): 30% of the source points. For each test case, 300 randomized trials were conducted, with successful registrations being used to compute an average target registration error (TRE). The error bars provide approximate standard deviations of the reported average TRE values. The proposed IMLP algorithm was evaluated relative to standard ICP [1], GICP [11], a robust variant of ICP [4], and CPD [20].

The TRE achieved by each algorithm in this study is presented in Fig. 6 for the range of small misalignments (Experiment 5A) and in Fig. 7 for the range of large misalignments (Experiment 5B). Figs. 6 and 7 are each divided into four sub-figures (A-D) corresponding to sub-experiments (i-iv) of Experiments 5A and 5B, respectively, for each level of outliers. As in the prior studies, the TRE outcomes for each misalignment range are very similar. As seen in the figures, IMLP achieves large improvement in registration accuracy relative to the other algorithms for up to 20% outliers, even in comparison to CPD, which has a very effective outlier rejection capability. For the 30% outlier case, IMLP continues to provide accurate results and compares approximately equal to CPD. Compared to Robust ICP, IMLP is substantially more accurate in all test cases and frequently achieves less than half the registration error and below. As expected, standard ICP and GICP perform poorly, since they are non-robust techniques and do not include mechanisms to account for outliers. Robust ICP fairs much better than the non-robust methods for outlier compositions of 10% and below, but produces higher registration error than standard ICP for the outlier percentages above 10%.

Table 10 shows the rate of registration failure for both ranges of misalignment. For small misalignment (Experiment 5A) all algorithms achieve very low failure rates for outlier compositions up to 20%, with exception of GICP which has high failure rate at 20% outliers and beyond. At 30% outliers, the failure rates of Robust ICP and IMLP moderately increase whereas the failure rates of ICP and CPD remain low with CPD achieving no registration failure. For large misalignment, the failure rates of all algorithms are increased, with CPD again providing the best performance. IMLP is approximately on-par with CPD for outliers up to 10%. At 20% outliers, the failure rate of IMLP increases to a moderate 2–6.7%; at 30% outliers, the failure rate increases significantly to 12% and beyond. In contrast, the Robust ICP algorithm performs poorly across the board with an average failure rate above 10% for all percentages of outliers.

**Table 10:** Registration failure rates for registering a point-cloud target shape with outliers. (Experiment 5).

**Exp.**	**Outliers**	**Alg.**	**Failure Rate (%) by Test Case**								
			**1**	**2**	**3**	**4**	**5**	**6**	**7**	**8**	**9**
5A-i	5%	ICP	0.0	0.0	0.0	0.0	0.0	0.0	0.0	0.0	0.0
		GICP	0.0	0.0	0.0	0.0	0.3	0.0	0.0	0.0	0.3
		Robust ICP	0.0	0.3	0.0	0.0	0.0	0.0	0.3	0.3	0.3
		CPD	0.0	0.0	0.0	0.0	0.0	0.0	0.0	0.0	0.0
		IMLP	0.0	0.0	0.0	0.0	0.0	0.0	0.0	0.0	0.0
5A-ii	10%	ICP	0.0	0.0	0.0	0.0	0.0	0.0	0.0	0.0	0.0
		GICP	0.0	1.3	1.3	0.7	1.0	0.3	0.7	1.0	1.7
		Robust ICP	0.3	0.0	0.0	0.0	0.3	0.3	1.0	0.3	0.3
		CPD	0.0	0.0	0.0	0.0	0.0	0.0	0.0	0.0	0.0
		IMLP	0.0	0.0	0.0	0.0	0.0	0.0	0.0	0.0	0.0
5A-iii	20%	ICP	0.3	0.0	0.0	0.3	0.3	0.0	0.0	0.0	0.3
		GICP	5.7	7.0	6.3	5.7	8.0	9.3	5.3	4.3	8.3
		Robust ICP	0.3	0.3	0.3	0.3	1.0	0.3	0.0	0.0	0.0
		CPD	0.0	0.0	0.0	0.0	0.0	0.0	0.0	0.0	0.0
		IMLP	0.0	0.3	0.0	1.0	0.3	1.0	1.7	0.7	0.0
5A-iv	30%	ICP	1.3	0.3	1.0	0.7	0.3	1.0	0.3	1.0	0.3
		GICP	17.0	13.3	18.7	15.7	21.3	15.3	14.3	18.0	15.7
		Robust ICP	4.7	2.0	3.3	2.3	3.0	4.7	1.0	1.3	1.7
		CPD	0.0	0.0	0.0	0.3	0.0	0.0	0.0	0.0	0.0
		IMLP	4.7	3.0	5.3	3.3	6.0	3.3	3.3	3.0	4.0
5B-i	5%	ICP	0.3	1.3	0.7	0.3	0.3	0.3	1.7	0.0	0.7
		GICP	1.3	3.0	1.0	1.7	1.0	2.0	1.7	1.0	1.0
		Robust ICP	14.3	9.7	8.3	12.7	15.0	11.3	11.0	12.7	8.0
		CPD	1.0	0.3	0.7	1.7	1.7	1.0	0.7	1.3	1.7
		IMLP	1.0	0.3	0.3	0.3	0.0	0.7	2.0	0.7	1.3
5B-ii	10%	ICP	1.0	0.3	0.3	1.7	1.0	0.7	0.3	0.7	0.7
		GICP	1.7	2.7	2.3	3.0	3.3	3.3	2.0	2.7	2.3
		Robust ICP	12.7	12.3	11.3	11.0	12.0	10.7	10.7	11.3	9.3
		CPD	1.0	1.7	1.7	0.7	1.0	1.7	1.3	0.7	0.7
		IMLP	1.0	0.3	1.3	2.0	0.7	1.0	1.3	0.7	1.0
5B-iii	20%	ICP	0.7	0.7	1.0	1.3	0.7	0.0	0.3	0.7	0.7
		GICP	5.0	5.0	8.0	9.0	9.7	8.3	8.7	5.3	7.3
		Robust ICP	10.0	7.0	9.7	13.7	11.0	14.7	12.7	10.0	11.3
		CPD	1.0	2.3	1.3	1.3	1.3	2.3	0.7	1.7	1.0
		IMLP	2.7	4.3	2.0	4.7	6.7	4.7	5.7	3.7	3.3
5B-iv	30%	ICP	2.7	1.0	1.7	1.3	1.3	1.3	2.0	2.0	0.7
		GICP	15.0	17.7	12.3	17.7	19.3	20.7	18.3	21.7	20.7
		Robust ICP	16.3	10.0	15.3	15.3	16.0	15.3	12.7	17.0	16.0
		CPD	2.0	3.3	3.3	3.7	1.7	2.0	2.0	1.7	2.3
		IMLP	18.7	12.0	16.0	16.7	19.0	20.7	15.3	16.7	15.7

Source shapes were randomly generated from a mesh model of a human hip (Fig. 1A), misaligned by [15, 30] mm / degrees in (Experiment 5A) and [30, 60] mm / degrees in (Experiment 5B), and registered back to a point-cloud representation of the mesh. The test cases represent the different noise models used to generate noise on the source shape (Table 4). Outliers were added to the source shape constituting 5% (-i), 10% (-ii), 20% (-iii), and 30% (-iv) of the source points. For each test case, 300 randomized trials were conducted with the percent of unsuccessful registrations (TRE > 10 mm) being shown in the table. The proposed IMLP algorithm was evaluated relative to standard ICP [1], GICP [11], a robust variant of ICP [4], and CPD [20].

### Experiment 6: Sub-Shape Registration

This study investigates the more challenging problem of registering sub-shape data to a complete-shape model, which arises when measurements are taken from a sub-region of a shape to be registered. This scenario is investigated by simulating random measurements from a sub-region of a proximal human femur that has been segmented from CT imaging to form a mesh of the bone surface (Fig. 1B).

The experimental procedure for this study parallels that of Experiment 4, except that the area sampled for the source shape is confined to a sub-region of the target, which is represented by the darkly shaded region of the mesh in Fig. 1B. Random misalignments for this study are generated on the interval [10, 20] mm translation and [10, 20] degrees rotation. The same algorithms as in Experiment 4 are evaluated using identical settings except that the maximum iteration count for CPD was increased to 200, as 100 iterations was insufficient (other algorithms did not require this increase).

In order to diversify the range of noise conditions considered overall, a different set of noise conditions was investigated for this study. Table 11 defines the seven noise models investigated, which includes the zero-noise case, two magnitudes of isotropic noise, two cases of surface-oriented noise to test high variance in both surface-normal and surface-parallel directions, and two test cases involving randomly oriented noise models applied at a global and at a per-point scale. For the test cases involving randomly oriented noise models, different covariances were randomly generated for each trial. As in the prior experiments, 300 randomized trials were conducted for each test case.

**Table 11:** Generative noise models (test cases) used in the randomized registration trials of Experiment 6.

**Noise Covariance**	**Test Case**						
	**1**	**2**	**3**	**4**	**5**	**6**	**7**
Orientation	-	-	-	Surface	Surface	Random-Global	Random-Per-Point
Magnitude: $\lambda_{1}^{1/2}$ (mm)	0.0	0.5	1.0	1.0	0.5	0.5	0.5
Magnitude: $\lambda_{2,3}^{1/2}$ (mm)	0.0	0.5	1.0	0.5	1.0	1.0	1.0

This table defines the covariances used to generate the zero-mean, multi-variate, Gaussian noise applied to the source shape in each test case of Experiment 6. The table defines both the eigenvalues (*λ*_1_, *λ*_2_, *λ*_3_) (magnitude) and the eigenvectors (orientation) of the covariance matrices for each test case. Orientation “Surface” defines the eigenvectors relative to the orientation of the surface at each source point, where *λ*_1_ is the variance in the surface-normal direction and (*λ*_2_, *λ*_3_) are the variances in the surface-parallel directions. Orientation “Random-Global” defines the matrix of eigenvectors to be a randomly generated rotation matrix, which is applied globally over the source shape (i.e. every point in the source shape has the same noise model). Orientation “Random-Per-Point” is similar to the “Random-Global” case except that every point in the source shape is associated with a different randomly generated rotation (i.e. every point in the source shape has a different noise model). Orientation “-” means that the noise model is isotropic and thus unaffected by the choice of eigenvectors.

**Fig 8:**
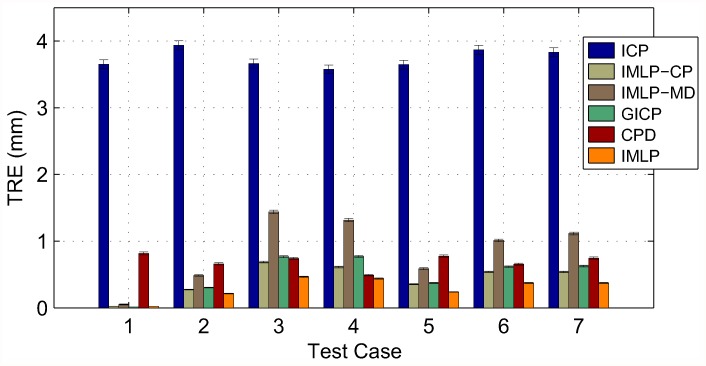
Registration errors for registering a sub-shape. (Experiment 6). Source shapes were randomly generated from a sub-region of a mesh model of a human proximal femur (Fig. 1B), misaligned by [10, 20] mm / degrees, and registered back to a point-cloud representation of the mesh. The test cases represent the different noise models used to generate noise on the source shape (Table 11). For each test case, 300 randomized trials were conducted, with successful registrations being used to compute an average target registration error (TRE). The error bars provide approximate standard deviations of the reported average TRE values. The proposed IMLP algorithm was evaluated relative to standard ICP [1], GICP [11], and CPD [20], as well as relative to near-comparisons of GTLS-ICP [10] and A-ICP [12] using the two variants IMLP-CP and IMLP-MD, which modify IMLP’s most-likely match criteria to that of closest-point and Mahalanobis-distance matching, respectively.

Fig. 8 shows the registration errors and Table 12 presents the registration failure rates achieved by each algorithm in this study. The standard ICP algorithm turns out poor performance across the board in terms of both registration error (above 3.5 mm) and failure rate (approximately 11–19%). CPD has the best success rate with almost no failures overall. The failure rates of other anisotropic methods are approximately on-par with the IMLP failure rate of 3–7%, with the IMLP-CP variant being marginally lower than the others. As seen in the figure, IMLP achieves the lowest registration error in every test case. Compared to CPD the improvement in registration error by IMLP is often substantial, especially for the zero-noise case where IMLP achieves nearly zero registration error and CPD has an average TRE near 1 mm. Again, we find that the Mahalanobis-distance match criterion, as assessed by IMLP-MD, computes substantially worse registration error than the closest-point match criterion, as assessed by IMLP-CP and as also used by GICP; on the other hand, the most-likely match criterion of IMLP achieves the lowest registration error in every case.

**Table 12:** Registration failure rates for sub-shape registration. (Experiment 6).

**Alg.**	**Failure Rate (%) by Test Case**						
	**1**	**2**	**3**	**4**	**5**	**6**	**7**
ICP	15.0	10.7	17.3	13.7	14.7	18.7	16.3
IMLP-CP	4.7	2	5.3	4.3	4.3	4.3	4.0
IMLP-MD	6.0	3.3	7.3	5.3	7.0	6.7	5.3
GICP	6.0	4.3	8.3	6.3	6.0	5.3	4.7
CPD	0.0	0.0	0.0	0.0	0.0	0.3	0.3
IMLP	6.0	3.0	7.0	5.0	6.0	6.3	5.0

Source shapes were randomly generated from from a mesh model of a human femur (Fig. 1B), misaligned by [10, 20] mm / degrees, and registered back to a point-cloud representation of the mesh. The test cases represent different noise models used to generate noise on the source shape (Table 11). For each test case, 300 randomized trials were conducted with the percent of unsuccessful registrations (TRE > 10 mm) being shown in the table. The proposed IMLP algorithm was evaluated relative to standard ICP [1], GICP [11], and CPD [20], as well as relative to near-comparisons of GTLS-ICP [10] and A-ICP [12] using the two IMLP variants IMLP-CP and IMLP-MD, which modify IMLP’s most-likely match criteria to that of closest-point and Mahalanobis-distance matching, respectively.

### Experiment 7: Registering Shapes with Partial Overlap

**Fig 9:**
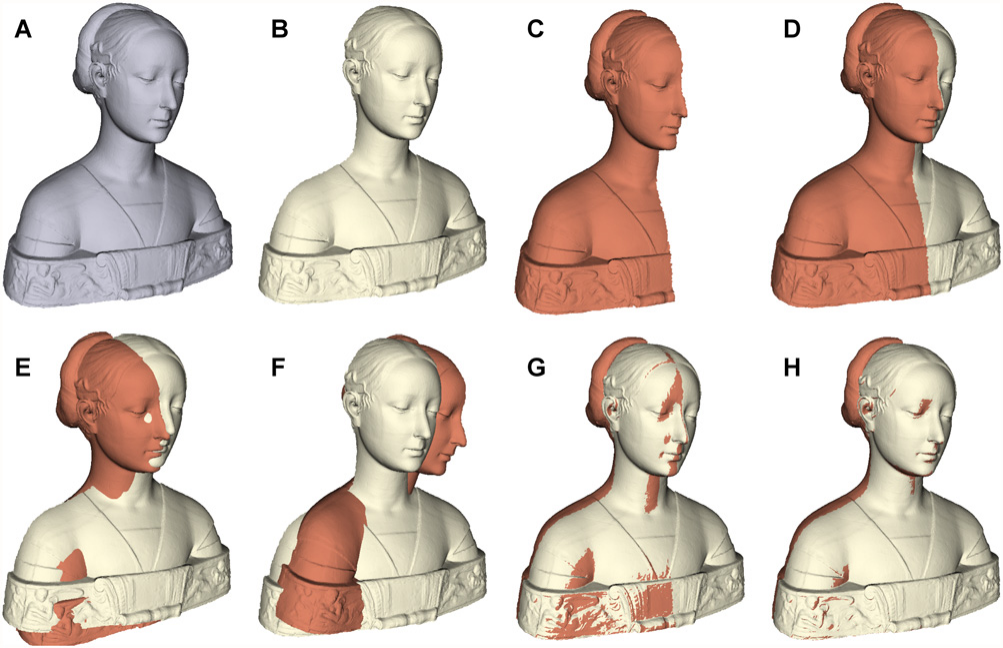
Registration of shapes having partial overlap. (Experiment 7). (A): The statue Laurana sub-divided into (B): front and (C): right half-sections, such that (D): a 50% overlap exists between the two sub-shapes. The sub-shapes were (E): misaligned by 10 mm and 10 degrees in a random direction and then registered using (F): CPD [20], (G): GICP [11], and (H): the proposed IMLP algorithm. Sub-figures (E-H) show the initial misalignment and the final registered alignments of the two shapes for the 6th randomized trial of Experiment 7, which involved 10 randomized trials in total.

This study investigates a yet more challenging problem of registering two shapes that have only partial overlap, meaning there are regions of both the source and target shape that do not have any true correspondence with the other shape. To investigate this scenario, we use a model of the statue Laurana (Fig. 9A) provided by the Institute of Science and Technologies (ISTI-CNR), Pisa, Italy, which was downloaded under a Creative Commons License from http://vcg.isti.cnr.it/downloads/3dgallery/form_laurana.htm. A decimation was applied to the original mesh using the Quadric Edge Collapse Decimation in MeshLab [42] to reduce the model to 50,000 triangles, and the coordinate system was adjusted to position the origin at the mesh centroid. Two divisions of the mesh were then performed to extract the front (Fig. 9B) and right (Fig. 9C) half-sections of the model. A dense point cloud for the source shape was formed from the vertices of the right half-section, and a dense point cloud for the target shape was defined from the center points of the triangles of the front half-section. Thus, the region of overlap between the source and target shapes comprised 50% of each shape (Fig. 9D).

Ten randomized registration trials were performed by applying 0.5 mm standard deviation of isotropic Gaussian noise to the source points and applying misalignments of 10 mm and 10 degrees in random directions. Validation points for computing the TRE were selected randomly from the target shape, not being confined to the region of overlap.

The algorithms evaluated in this study were GICP [11], CPD [20], and IMLP. Various values were experimentally tested for the match threshold distance of GICP, with 3 mm finally selected as having the lowest TRE. For CPD, various outlier weights were tested with poor results obtained in every case; we therefore applied the standard outlier weight of 0.5. For IMLP, we used the default chi-square inverse CDF threshold ($\chi_{\text{thresh}}^{2}$) value of 7.81. To minimize bias from the non-overlapping region, matches identified as outliers were configured to be completely disregarded in the registration phase of the IMLP algorithm (rather than inflating their variances), as suggested in the Methods section for this type of application. The measurement-error covariances of IMLP were set to zero in this study in order to, as much as possible, enable the noise model to adapt to the region of overlap based on the match uncertainty term. To restrict “good” matches to the region of overlap, the max threshold for the match uncertainty parameter ($\sigma_{\text{max}}^{2}$) was set to 0.1 mm^2^. The surface-model covariances for both GICP and IMLP were set to the same values as used in Experiment 4.

Table 13 shows the TREs computed by each algorithm for each of the 10 randomized trials. As indicated in the table, CPD is unable to properly register any of the trials in this scenario, whereas both IMLP and GICP register all of them nearly perfectly, with GICP having a moderate to slight accuracy advantage. The bottom row of Fig. 9 provides a visualization of the initial shape misalignment (Fig. 9E) and of the registered alignments computed by each algorithm, including CPD (Fig. 9F), GICP (Fig. 9G), and IMLP (Fig. 9H). The visualizations of Figs. 9E-H represent the 6th randomized registration trial of this experiment.

**Table 13:** Registration errors for registering shapes having partial overlap. (Experiment 7).

**Alg.**	**TRE (mm) per Registration Trial**									
	**1**	**2**	**3**	**4**	**5**	**6**	**7**	**8**	**9**	**10**
CPD	63.533	69.988	82.186	83.449	72.612	71.378	69.431	79.822	78.071	68.675
GICP	0.138	0.150	0.187	0.060	0.124	0.165	0.272	0.252	0.158	0.242
IMLP	0.264	0.306	0.283	0.224	0.352	0.294	0.295	0.277	0.250	0.365

Target registration error (TRE) is reported for each of 10 registration trials conducted for Experiment 7, which involve registering two half-sections of the statue Laurana that have 50% true overlap (Fig. 9D). The half-sections were randomly misaligned by 10 mm and 10 degrees and then registered using CPD [20], GICP [11], and the proposed IMLP algorithm.

### Experiment 8: Runtime Comparison of Methods for Computing the Most-Likely Matches

As a final study, an investigation is made concerning the speedup afforded by the PD-tree search technique used to compute the most-likely matches for IMLP. We compare the runtime of a naive exhaustive search to that of the proposed PD-tree method using both the spherical (14) and simple ellipsoidal (15) bounding techniques, as described in the Methods section.

Table 14 shows the average runtimes obtained from running IMLP over all 300 trials of Test Case 1 from Experiment 4B for each method of computing the most-likely matches. As seen in the table, the proposed PD-tree strategy achieves more than 140x speedup over the naive search when using the simple ellipsoidal bounding technique. Comparing the two alternative PD-tree bounding methods indicates that the more compact simple ellipsoidal bound achieves approximately 10% greater runtime efficiency than the spherical bound, even though its computations are significantly more complex.

**Table 14:** IMLP runtime comparison using different PD-tree bounding methods for computing the most-likely matches.

**Search Method**	Naive Search	PD-Tree: Spherical Bound	PD-Tree: Simple Ellipsoidal Bound
**Runtime (sec.)**	18.523	0.141	0.128

Average runtimes are reported for the proposed IMLP algorithm over the 300 registration trials of Experiment 4B, Test Case 1, which involves registering 100 random samples to a point-cloud representation of a hip model (Fig. 1A). Runtimes were recorded for the naive exhaustive search and for the proposed PD-tree search strategy comparing two of the proposed PD-tree bounding methods: the spherical (14) and simple ellipsoidal (15) bounds. The compact ellipsoidal bound (17) was not evaluated.

We note that the most compact ellipsoidal bound (17), which is not evaluated here, may enable even further speedup over the simple ellipsoidal bound evaluated above. This is a likely outcome, since the runtime computations performed for each ellipsoidal bounding technique are very similar; thus, the most compact bound should provide the best performance.

## Conclusions

We have presented a novel variant of the Iterative Closest Point (ICP) algorithm, called the Iterative Most-Likely Point (IMLP) algorithm, which has the ability to compute optimal shape alignment under anisotropic noise conditions by incorporating a probabilistic framework within both the correspondence and registration phases of the algorithm. Another advantage of this framework is the ability to model locally-linear regions of a continuous surface, as also done by prior methods, which greatly improves the registration accuracy attainable from discrete representations of a surface. Dynamic estimation of the match uncertainty enables IMLP to adaptively adjust its noise model to different levels of misalignment, which provides robustness under large initial misalignments and high accuracy and sensitivity to outliers when in the vicinity of the correct solution. In addition, the probabilistic underpinning provides a cohesive and flexible framework for detection and mitigation of outliers, as well as enabling registration of shapes having only partial overlap via a user-defined maximum threshold on the match uncertainty term.

Through an extensive set of experiments, involving more than 50,000 randomized executions of the IMLP algorithm alone, IMLP has been shown to possess significant registration accuracy and robustness advantages compared to long-established and recently introduced algorithms over a broad range of test conditions including various noise conditions, percentages of outliers, ranges of misalignment, and test shapes. Other algorithms evaluated include the long-established algorithm of standard ICP [1] and a robust ICP variant [4], as well as the more recent, leading algorithms of GICP [11] and CPD [20]. In addition, close comparison is made to the prior anisotropic registration methods of GTLS-ICP [10] and A-ICP [12] using modifications on our own method, IMLP-CP and IMLP-MD, respectively. Relative to all tested algorithms, IMLP demonstrated a clear accuracy advantage overall.

Compared to CPD, which has a very effective outlier mitigation capability, IMLP was demonstrated to achieve equivalent registration success rates for outlier percentages of 10% and below, with marginal to moderate relative increase in failure rate at 20% outliers and large relative increase at 30% outliers. On the other hand, in terms of the registration accuracy of successful trials, IMLP achieves significantly better or on-par accuracy compared to CPD for all levels of outliers studied (up to 30%). Based on our results, we conclude that IMLP is a very effective method for registering shapes with up to 10% outliers and retains excellent performance at 20% outliers for moderate levels of misalignment.

Only for the experiment involving registration of partially-overlapping shapes did another algorithm (GICP) clearly come ahead of IMLP in terms of registration accuracy. However, IMLP nonetheless demonstrated a strong performance in this scenario and achieved higher accuracy than GICP in all other experiments performed. Further, the CPD algorithm failed completely in this scenario.

A surprising outcome of our experiments reveal that the Mahalanobis-distance match criterion consistently performs worse than the closest-point match criterion for registrations involving point-cloud targets, whereas for the case of a mesh target the opposite is true. In contrast, the most-likely match criterion of IMLP provides the best performance in both scenarios. These observations were evaluated using two variants of IMLP—IMLP-CP and IMLP-MD— which incorporate the modified match criteria of closest-point and Mahalnobis-distance matching, respectively, all else being equal.

Although IMLP is several times slower than standard ICP, it nonetheless provides a very competitive runtime, considering the substantial reduction in registration error that it achieves. Compared to CPD (the next-best performing algorithm overall), IMLP achieves better registration accuracy in the majority of test cases considered, while being approximately two orders of magnitude more efficient. While IMLP is efficient enough to run on its own, further substantial speed-up could be easily obtained by initializing the registration with a faster algorithm such as standard ICP, as demonstrated in prior work [12]. Furthermore, the computations performed by IMLP are highly parallelizable and may be efficiently implemented on a GPU as also demonstrated in prior work regarding ICP-based algorithms [43]. Finally, since we have used the simple ellipsoidal bounding method (15) for the PD-tree search in our implementation, further speedup may be possible by implementing the more compact ellipsoidal bound of (17).

As alluded to in the foregoing paragraph, we have also presented in this paper an effective and novel search strategy for computing the most-likely matches on a target shape with respect to IMLP’s most-likely match criterion. As demonstrated in our results, the proposed search strategy provides a massive speedup (>140x) over a naive search. This speedup is a key enabler of the efficient runtime performance achieved by IMLP. While this search strategy was devised to compute point correspondences based on the most-likely match criterion of IMLP, our method is equally applicable to the anisotropic Mahalanobis-distance match criterion of A-ICP.

In this paper, we have also presented an alternative approach for solving the generalized total-least-squares (GTLS) problem of aligning two corresponding point sets under a generalized noise model. The proposed approach turns out to be that of a Gauss-Newton-based method, which we have demonstrated to be more accurate, efficient, and stable compared to prior solutions proposed for this problem. The proposed approach supports anisotropic error in both sets of points being registered and is easily implemented using a standard least-squares solver, which avoids the software dependency of a nonlinear optimization library. In addition to its incorporation within the IMLP algorithm, our GTLS registration approach may also be used to implement related algorithms that incorporate generalized noise models, such as GICP.

In future work, we will investigate use of the compact ellipsoidal bounding method (17) for the PD-tree search to determine what added speedup may be gained by incorporating this improved bound. It will also be interesting to further investigate the problem of registering partially-overlapping shapes to determine how the performance of IMLP may be improved to that of GICP for this particular scenario.

## Supporting Information

S1 Appendix Computing the Point of Most-Likely Correspondence on a PD-Tree Datum. (PDF)

S2 Appendix Equivalent Forms for the Generalized Total-Least-Squares (GTLS) Problem of Aligning Corresponding Point Sets. (PDF)
