# Supplementary material for: Iterative Most-Likely Point Registration (IMLP): A Robust Algorithm for Computing Optimal Shape Alignment
Source: PLoS One. 2015 Mar 6;10(3):e0117688. doi: 10.1371/journal.pone.0117688 (PMC4352012; doi:10.1371/journal.pone.0117688)
Supplement: S1 Appendix — (PDF) [file pone.0117688.s001.pdf]

S1 Appendix: Computing the Point of Most-Likely Correspondence on a PD-Tree Datum

In this appendix we briefly discuss how to compute the most-likely correspondence for a given datum of a PD-tree, as required in Step 6 of Algorithm 4 for performing the PD-tree search described in the Methods section of the main manuscript. Performing this computation is required when searching a leaf node of the tree, where a match error is computed for each datum within the leaf node.

When the target shape is represented by a point cloud, computing the most-likely correspondence point on a datum is trivial, since each datum consists of only a single point, y. In this case, each datum has associated with it a noise-model covariance, M_y, that is stored in the PD tree alongside the datum point. The match error for the datum is then simply computed as

$$\mathrm{E}_{\mathrm{match}}(\vec{x}, \vec{y}, M_x, M_y, R, \vec{t}) = \log|RM_xR^T + M_y| + (\vec{y} - R\vec{x} - \vec{t})^T(RM_xR^T + M_y)^{-1}(\vec{y} - R\vec{x} - \vec{t}) \quad (1)$$

where R and $\vec{t}$ are the current estimates of the transformation parameters and where x and M_x represent the source point being matched and its associated noise-model covariance.

The problem is more complicated when the target shape is represented by a mesh, where each datum represents a single triangle in the mesh. For the experiments in this paper, we make the assumption that all points on a given triangle share a common noise model. Thus, a single covariance, M_y, is associated with each datum triangle and stored in the PD tree.

The most-likely match on a triangular datum is computed by performing a transformation of the physical space in order to transform the ellipsoidal level sets of the match-error function to a new space where the match-error function has spherical level sets. In this new space, the best match is then simply computed as the closest point on the transformed triangle to the source point. The procedure is to first transform each vertex $\vec{v_i}$ of the datum triangle to form a new triangle having vertices $\vec{v_i'}$

$$\vec{v_i'} = N(\vec{v_i} - R\vec{x} - t) \quad \text{for } i = 1, \ldots, n \quad (2)$$

where N is defined as

$$NN^T = (RM_xR^T + M_y)^{-1}\,. \quad (3)$$

The second step is to compute the closest point on the new triangle to the origin; we refer to this point

as $\vec{y}'$. The final step is to transform $\vec{y}'$ back to the original space by applying the inverse operation of (2), which provides the most-likely match $\vec{y}$ for this datum. The match error of the datum is then computed by (1) using the most-likely match point, $\vec{y}$, and the covariance associated with the datum triangle.
