# Supplementary material for: Iterative Most-Likely Point Registration (IMLP): A Robust Algorithm for Computing Optimal Shape Alignment
Source: PLoS One. 2015 Mar 6;10(3):e0117688. doi: 10.1371/journal.pone.0117688 (PMC4352012; doi:10.1371/journal.pone.0117688)
Supplement: S2 Appendix — (PDF) [file pone.0117688.s002.pdf]

S2 Appendix: Equivalent Forms for the Generalized Total-Least-Squares (GTLS) Problem of Aligning Corresponding Point Sets

In this appendix, we establish the equivalence between two different representations for the generalized total-least-squares (GTLS) problem of registering two corresponding point sets under a generalized noise model. Our aim is to show that the unconstrained optimization

$$\mathrm{E}_{\mathrm{GTLS}}(X, Y, M_X, M_Y) = \min_{R,\vec{t}} \sum_{i=1}^{n} \left(\vec{y_i} - R\vec{x_i} - \vec{t}\right)^T \left(RM_{\mathrm{x}i}R^T + M_{\mathrm{y}i}\right)^{-1} \left(\vec{y_i} - R\vec{x_i} - \vec{t}\right) \tag{1}$$

is equivalent to the constrained optimization

$$\begin{gathered}\mathrm{E}_{\mathrm{GTLS}}(X, Y, M_X, M_Y) = \min_{R,\vec{t}} \sum_{i=1}^{n} \left(\vec{x_i} - \vec{x_i}^*\right)^T M_{\mathrm{x}i}^{-1} \left(\vec{x_i} - \vec{x_i}^*\right) + \sum_{i=1}^{n} \left(\vec{y_i} - \vec{y_i}^*\right)^T M_{\mathrm{y}i}^{-1} \left(\vec{y_i} - \vec{y_i}^*\right) \\ \text{subject to: } \vec{y_i}^* = R\vec{x_i}^* - \vec{t}\end{gathered} \tag{2}$$

where $X = \{\vec{x_i}\}$ and $Y = \{\vec{y_i}\}$ are the measured source and target point sets and $M_X = \{M_{\mathrm{x}i}\}$ and $M_Y = \{M_{\mathrm{y}i}\}$ are the noise covariances of the measured points. The point sets $\{\vec{x_i}^*\}$ and $\{\vec{y_i}^*\}$ represent the optimizer's estimates for the unknown, noise-free positions of the source and target points which, due to the correspondence assumption, are constrained to have perfect alignment under the transformation parameters, R and $\vec{t}$, that are being solved by the optimization.

To establish an equivalence between (1) and (2), we begin at (2) by deriving expressions for the estimates of the noise-free point sets $\{\vec{x_i}^*\}$ and $\{\vec{y_i}^*\}$ in terms of the measured points, noise covariances, and transformation parameters. These expressions will then be substituted into the cost function of (2) which, through a subsequent series of algebraic simplifications, will be shown to be equivalent to the form of (1).

To begin, we solve for expressions of the noise-free estimates $\{\vec{x_i}^*\}$ and $\{\vec{y_i}^*\}$. This may be accomplished using the method of Lagrange multipliers. Redefining the constraints as

$$\mathrm{F}_i(\vec{x_i}^*, \vec{y_i}^*, R, \vec{t}) = \vec{y_i}^* - R\vec{x_i}^* - \vec{t} = 0 \tag{3}$$

we obtain the following Lagrangian function

$$\mathcal{L} = \sum_{i=1}^{n} (\vec{x_i} - \vec{x_i}^*)^T M_{\mathrm{x}i}^{-1} (\vec{x_i} - \vec{x_i}^*) + \sum_{i=1}^{n} (\vec{y_i} - \vec{y_i}^*)^T M_{\mathrm{y}i}^{-1} (\vec{y_i} - \vec{y_i}^*) + \lambda_i^T \, \mathrm{F}_i(\vec{x_i}^*, \vec{y_i}^*, R, \vec{t}) \tag{4}$$

which may be expressed in matrix form as

$$\mathcal{L} = (X - X^*)^T M_X^{-1} (X - X^*) + (Y - Y^*)^T M_Y^{-1} (Y - Y^*) + \lambda^T F \tag{5}$$

where we have defined

$$X = \begin{bmatrix} \vec{x_1} \\ \vdots \\ \vec{x_n} \end{bmatrix}, \quad X^* = \begin{bmatrix} \vec{x_1^*} \\ \vdots \\ \vec{x_n^*} \end{bmatrix}, \quad Y = \begin{bmatrix} \vec{y_1} \\ \vdots \\ \vec{y_n} \end{bmatrix}, \quad Y^* = \begin{bmatrix} \vec{y_1^*} \\ \vdots \\ \vec{y_n^*} \end{bmatrix},$$

$$M_X = \begin{bmatrix} M_{\mathrm{x}1} & & \\ & \ddots & \\ & & M_{\mathrm{x}n} \end{bmatrix}, \quad M_Y = \begin{bmatrix} M_{\mathrm{y}1} & & \\ & \ddots & \\ & & M_{\mathrm{y}n} \end{bmatrix}, \quad \lambda = \begin{bmatrix} \lambda_1 \\ \vdots \\ \lambda_n \end{bmatrix}, \quad F = \begin{bmatrix} \mathrm{F}_i(\vec{x_1}^*, \vec{y_1}^*, R, \vec{t}) \\ \vdots \\ \mathrm{F}_i(\vec{x_n}^*, \vec{y_n}^*, R, \vec{t}) \end{bmatrix}.$$

The next step is to minimize the Lagrangian function with respect to the estimates of the noise-free point sets. This is done by solving for the partial derivatives of the Lagrangian function with respect to each estimate

$$\frac{\partial \mathcal{L}}{\partial X^*} = -2M_X^{-1}(X - X^*) - \mathrm{diag}(R^T)\lambda \tag{6}$$

$$\frac{\partial \mathcal{L}}{\partial Y^*} = -2M_Y^{-1}(Y - Y^*) + \lambda \tag{7}$$

and setting the partial derivatives to zero which leads to the following equations

$$(X - X^*) = -\frac{1}{2} M_X \, \mathrm{diag}(R^T)\lambda \tag{8}$$

$$(Y - Y^*) = \frac{1}{2} M_Y \lambda \,. \tag{9}$$

We now have all the equations required to solve for expressions of $\vec{x_i}^*$ and $\vec{y_i}^*$ in terms of the other parameters. Solving (9) for λ_i

$$\lambda_i = 2M_{\text{y}i}^{-1}\left(\vec{y_i} - \vec{y_i}^*\right) \tag{10}$$

and substituting into the relevant sub-component of (8) we obtain

$$\left(\vec{x_i} - \vec{x_i}^*\right) = -M_{\text{x}i}R^T M_{\text{y}i}^{-1}\left(\vec{y_i} - \vec{y_i}^*\right) \,. \tag{11}$$

Substituting the constraint from (2) into (11) we have

$$\left(\vec{x_i} - \vec{x_i}^*\right) = -M_{\text{x}i}R^T M_{\text{y}i}^{-1}\left(\vec{y_i} - R\vec{x_i}^* - t\right) \,. \tag{12}$$

Rearrangement of (12) produces the following expression for $\vec{x_i}^*$

$$\vec{x_i}^* = \left(I + M_{\text{x}i}R^T M_{\text{y}i}^{-1}R\right)^{-1}\left(\vec{x_i} + M_{\text{x}i}R^T M_{\text{y}i}^{-1}\left(\vec{y_i} - t\right)\right) \tag{13}$$

$$= \vec{x_i} + M_{\text{x}i}R^T\left(M_{\text{y}i} + RM_{\text{x}i}R^T\right)^{-1}\left(\vec{y_i} - R\vec{x_i} - t\right) \,. \tag{14}$$

The derivation of (14) from (13) is accomplished by expanding the multiplication with the inverse expression and applying the following helpful identities

$$(I + AB)^{-1} = I - A(BA + I)^{-1}B \tag{15}$$

$$(A + B)^{-1}C = (C^{-1}A + C^{-1}B)^{-1} \tag{16}$$

$$C(A + B)^{-1} = (AC^{-1} + BC^{-1})^{-1} \,. \tag{17}$$

The identity of (15) follows as a simplification of

$$(A + BCD)^{-1} = A^{-1} - A^{-1}B(DA^{-1}B + C^{-1})^{-1}DA^{-1} \,, \tag{18}$$

which is described in [1], while those of (16) and (17) are easily verified.

An expression for $\vec{y_i}$ is obtained in similar manner by solving (8) for λ_i and substituting into (9) along

with the constraint of (2), which leads to the equations

$$\vec{y_i}^* = \left(I + M_{\text{y}i}RM_{\text{x}i}^{-1}R^T\right)^{-1}\left(\vec{y_i} + M_{\text{y}i}RM_{\text{x}i}^{-1}\left(\vec{x_i} + R^T t\right)\right) \tag{19}$$

$$= \vec{y_i} - M_{\text{y}i}\left(RM_{\text{x}i}R^T + M_{\text{y}i}\right)^{-1}\left(\vec{y_i} - R\vec{x_i} - t\right) . \tag{20}$$

The next step is to substitute the expressions of $\vec{x_i}$ from (14) and of $\vec{y_i}$ from (20) into the cost function of (2). To simplify the resulting equations, we make the following definitions

$$M_i = \left(RM_{\text{x}i}R^T + M_{\text{y}i}\right)$$
$$\vec{d_i} = \vec{y_i} - R\vec{x_i} - \vec{t}.$$

Applying these substitutions, the two terms within the cost function of (2) become

$$(\vec{x_i} - \vec{x_i}^*)^T M_{\text{x}i}^{-1}(\vec{x_i} - \vec{x_i}^*) = \vec{d_i}^T M_i^{-1} RM_{\text{x}i}R^T M_i^{-1}\vec{d_i} \tag{21}$$

$$(\vec{y_i} - \vec{y_i}^*)^T M_{\text{y}i}^{-1}(\vec{y_i} - \vec{y_i}^*) = \vec{d_i}^T M_i^{-1} M_{\text{y}i} M_i^{-1}\vec{d_i} . \tag{22}$$

Substituting these equations into the cost function of (2), we have

$$\begin{aligned}
\text{E}_{\text{GTLS}}(X, Y, M_X, M_Y) &= \min_{R,\vec{t}} \sum_{i=1}^{n} \left[(\vec{x_i} - \vec{x_i}^*)^T M_{\text{x}i}^{-1}(\vec{x_i} - \vec{x_i}^*) + (\vec{y_i} - \vec{y_i}^*)^T M_{\text{y}i}^{-1}(\vec{y_i} - \vec{y_i}^*)\right] \\
&\qquad \text{subject to: } \vec{y_i}^* = R\vec{x_i}^* - \vec{t} \\
&= \min_{R,\vec{t}} \sum_{i=1}^{n} \left[\vec{d_i}^T M_i^{-1} RM_{\text{x}i}R^T M_i^{-1}\vec{d_i} + \vec{d_i}^T M_i^{-1} M_{\text{y}i} M_i^{-1}\vec{d_i}\right] \\
&= \min_{R,\vec{t}} \sum_{i=1}^{n} \vec{d_i}^T \left(M_i^{-1} RM_{\text{x}i}R^T M_i^{-1} + M_i^{-1} M_{\text{y}i} M_i^{-1}\right)\vec{d_i} \\
&= \min_{R,\vec{t}} \sum_{i=1}^{n} \vec{d_i}^T \left(M_i^{-1}\left(RM_{\text{x}i}R^T + M_{\text{y}i}\right) M_i^{-1}\right)\vec{d_i} \\
&= \min_{R,\vec{t}} \sum_{i=1}^{n} \vec{d_i}^T \left(M_i^{-1} M_i M_i^{-1}\right)\vec{d_i} \\
&= \min_{R,\vec{t}} \sum_{i=1}^{n} \vec{d_i}^T M_i^{-1}\vec{d_i} \\
&= \min_{R,\vec{t}} \sum_{i=1}^{n} \left(\vec{y_i} - R\vec{x_i} - \vec{t}\right)^T \left(RM_{\text{x}i}R^T + M_{\text{y}i}\right)^{-1}\left(\vec{y_i} - R\vec{x_i} - \vec{t}\right) \qquad (23)
\end{aligned}$$

where the final form of (23) is equivalent to (1), which completes the derivation of equivalence between (1) and (2) that we aimed to show.

References

1. Kailath T (1980) Linear systems. Prentice-Hall Englewood Cliffs, NJ.
